# Neuropeptide TIP39 as a pluripotent brain molecule and its potential functions in postpartum depression

**DOI:** 10.3389/fendo.2025.1674213

**Published:** 2026-01-08

**Authors:** Silin Wang, Hongchi Liu, Qiuyi Tang, Ning Liao, Tianhao Liu, Zhengzhong Yang, Yanghui Wei, Rao Fu

**Affiliations:** 1 Department of Anatomy, School of Medicine, Shenzhen Campus of Sun Yat-sen University, Sun Yat-sen University, Shenzhen, Guangdong, China; 2 Department of Surgery, The Eighth Affiliated Hospital, Sun Yat-sen University, Guangzhou, China; 3 Shenzhen Key Laboratory for Systems Medicine in Inflammatory Diseases, School of Medicine, Shenzhen Campus of Sun Yat-Sen University, Sun Yat-Sen University, Shenzhen, Guangdong, China

**Keywords:** the tuberoinfundibular peptide of 39 residues, PTH2, PTH2R, postpartum depression, hypothalamic–pituitary axis

## Abstract

**Background:**

The tuberoinfundibular peptide of 39 residues (TIP39) is a small peptide first extracted in 1997, which has garnered limited research attention. It belongs to the parathyroid hormone (PTH)–related peptides family and specifically binds the parathyroid receptor type 2 (PTH2R). It is gaining attention for its roles in mood regulation, maternal behavior, and social functions. Its potential in modulating these processes possibly positions TIP39 as an underexplored candidate for exploring complex physiological functions of postpartum depression.

**Aims:**

Given the accumulating evidence of TIP39’s involvement in various regulatory functions, this review aims to investigate its potential as a common pathway for postpartum depression PPD-related symptoms, including depressive mood and maternal behaviors. Specifically, the focus is on exploring the possibility of TIP39 as a key modulator in the pathophysiology of PPD, with the goal of identifying a new entry point for the treatment of this mental health disorder.

**Conclusions:**

The multifunctional nature of TIP39, as evidenced by its roles in diverse physiological processes, opens up a promising avenue for further investigation. The investigation of TIP39’s role in mood regulation and maternal behavior provides a possible rationale for its consideration as a therapeutic target for PPD. This review is a tentative step to bridge TIP39 and PPD symptoms, with the ultimate goal of providing a new sight for the remedy of this prevalent and often debilitating disorder.

## Highlights

- TIP39 shows conserved brain localization in emotion-related regions across species.
- The TIP39-PTH2R system supports maternal behavior and its deficiency may contribute to depression and care deficits.
- TIP39’s N - terminus is key for receptor selectivity and bioactivity.
- TIP39 is a critical but under-characterized regulator in postpartum depression, requiring systematic exploration of its therapeutic potential.

## 1 Introduction

The tuberoinfundibular peptide of 39 residues (TIP39) is a neuropeptide with 39 residues in length. It belongs to the parathyroid-related peptide family, which additionally contains parathyroid hormone (PTH), PTH-related peptides (PTHrP), and PTH-like hormones (PTHLH). Based on the similarity of TIP39 to PTH in its backbone structure and its activation of the parathyroid receptor type 2 (PTH2R), TIP39 is also defined as parathyroid hormone 2, or abbreviated as PTH2, in the UniGene database. This PTH2R-selective ligand is the endogenous ligand that most robustly and specifically activates PTH2R (1).Experiments have revealed the N-terminal of TIP39 as the determinant for its activity, since N-terminal truncation binds with 70-fold lower affinity to the PTH2 receptor than full-length TIP39. Moreover, deletion of six N-terminal residues converts it into a potent PTH1R antagonist (2, 3).

TIP39 was first purified in HEK293 cells from an acid extract of bovine hypothalamus and then was sequenced (4). It has been studied across various animal models, including rodents (4), primates like macaques (5), and non-mammal animals like zebrafish (6). Despite species differences, the spatial distribution of TIP39 is generally consistent, concentrated in the Periventricular Gray of the Thalamus (PVG), the Posterior Intralaminar Complex of the Thalamus (PIL), and the Medial Paralemniscal Nucleus (MPL) (7).

TIP39 has been shown to modulate the stress response by regulating the hypothalamic–pituitary axis (HPA) and processes nociceptive signals, as well as controlling body temperature (1). Injecting TIP39 into the brains of CUMS rats lessens the ratio of glucocorticoid and mineralocorticoid receptors (MRs) in the brain. TIP39 may be involved in modulating affective behaviors, especially negative emotions like anxiety (8) and depression (9), and social intercourse (10), by down-regulating glutamate and acetylcholinesterase levels while upregulating GABA levels (11). Some animal experiments have indicated great connection between the TIP39 levels and depression. Data shows that TIP39 level is inversely linked to depression-like behaviors in animals (9).

The involvement of the TIP39-PTH2R system in maternal behavior has been experimentally evaluated by Gellen et al. The experiment showed that PTH2R knockout (PTH2R-KO) female parental mice showed significantly higher depressive mood and less maternal behavior compared to wild type, indicating that the PTH2R system deficiencies are most likely involved in maternal depressive-like behavioral changes (12). TIP39 peptide levels significantly stimulate maternal behavior and vice versa (12, 13).

Taken together, TIP39-PTH2R signaling has shown improvements in each respective PPD-related symptom in rodent animal models via its great participation in mood elevation, stress regulation, and maternal behaviors (1, 12); yet, the overall combined effect hasn’t been elucidated. To further explore, experiments on PPD animal models are expected.

This review recapitulates the existing knowledge literature regarding TIP39 and attempts to bridge actions of TIP39 in the central nervous system (CNS) and postpartum depression.

## 2 Literature search methodology

This review followed the PRISMA guidelines. The final search was performed on 18 September 2025, covering the period from November 1999 to April 2025. The primary search for article screening used in this review was conducted using PubMed (*n* = 290), Embase (*n* = 139), Web of Science (*n* = 124), and Science direct (*n* = 238), and the medical subject headings (TIP39, PTH2R, brain functions, maternal behaviors, and postpartum depression).

The literature screening was conducted collaboratively by four researchers. Articles were screened based on their titles and abstracts, followed by a full-text assessment for eligibility.

The inclusion criteria were as follows:

- Original research articles, systematic literature reviews (SLRs), and meta-analyses (MA).
- Reviews focusing on TIP39 and/or PTH2R.
- Writing in English.

Articles on the following topics were excluded:

- Duplicate reports of the same study (when several reports of a study exist in different journals, the most complete version of the study was included).
- Conference abstracts.
- Informal literature surveys (no defined research questions; no defined search process; no defined data extraction process).
- Studies not directly relevant to the core focus of this review.

Following a rigorous selection process, 62 articles were included in our study.

**Figure 1:**
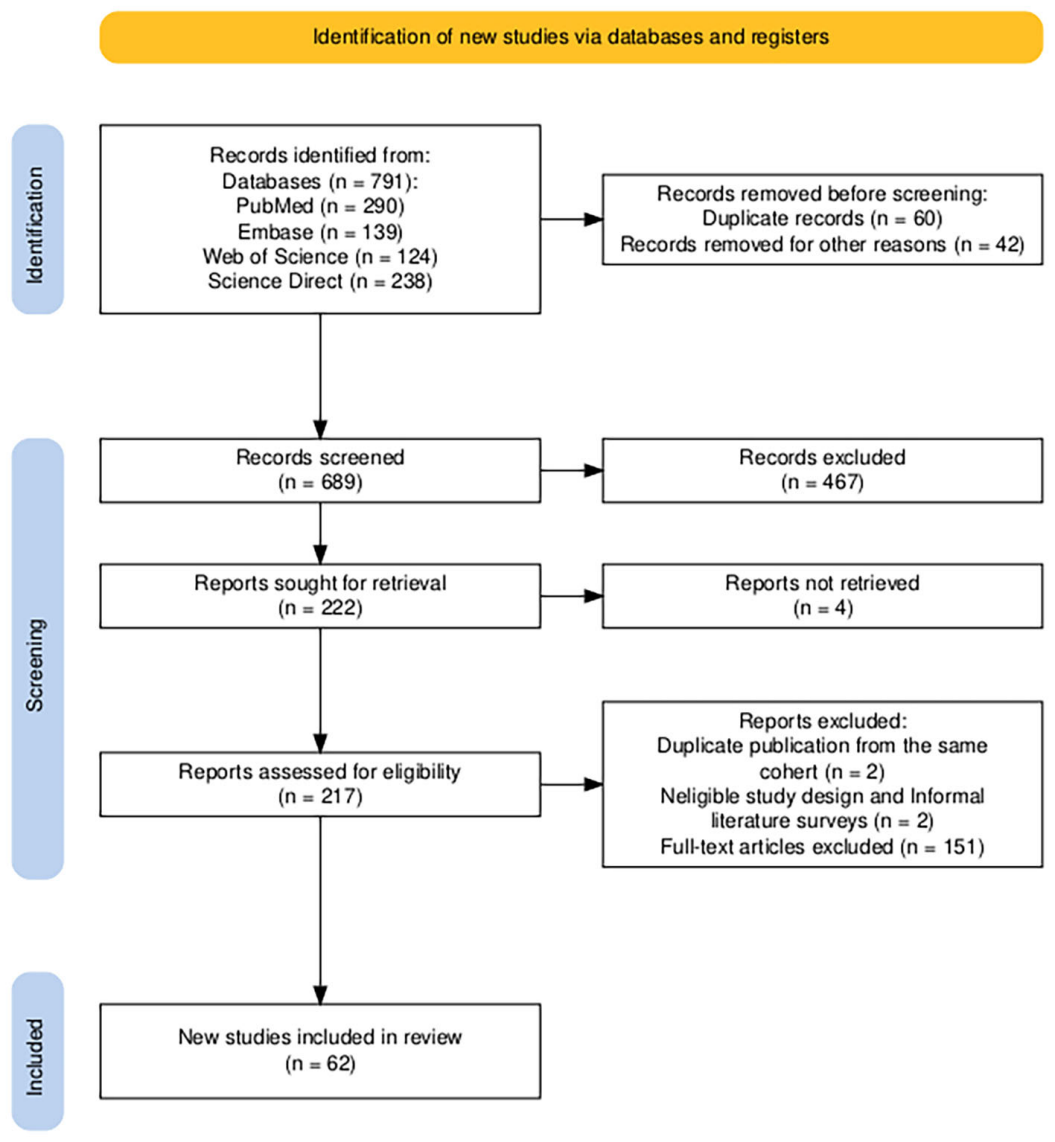
Flow PRISMA diagram representing the selection process of eligible studies, using PRISMA2020 tool (Haddaway, N. R., 2020).

A PRISMA flow chart outlining the study selection process is depicted in **Figure 1**.

## 3 Molecular characterization of TIP39

### 3.1 Structure

As the PTH2R agonist, a new neuropeptide, TIP39, initially purified from bovine hypothalamus, was identified through amino acid sequencing using Edman degradation on a microscale and confirmed by mass spectrometry. The sequence of the peptide is SLALADDAAFRERARLLAALERRHWLNSYMHKLLVLDAP (4, 14). There is limited identity existing between TIP39 and the related signaling peptide families PTH or PTHrP (nine and seven residues identical, respectively). When considering similar residues, the similarity of these three ligands is approximately 50% (15). Furthermore, TIP39 positions Asp7 in the critical site typically occupied by PTH (Ile5) and PTHrP (His5), making it perfectly fit into the binding pocket of PTH2R (16). The residue likely involved in this interaction is Tyr-318 of PTH2R (17).

The bovine TIP39 structure consists of two distinct α-helices, spanning residues Ala5–Arg21 and Leu26–Val35, with an unstructured region between them (16). High-resolution NMR studies suggest that TIP39 shares a parallel 3D backbone compared to PTH (18, 19). Furthermore, an analysis of the structural attributes of TIP39 and PTH reveals a comparable configuration of the N-terminal helix, which is pivotal in elucidating its activation of PTH2R (16).

Recent studies have reported the cryo-EM structure of the TIP39–PTH2R–Gs complex. TIP39 binding to PTH2R is stabilized by key interactions with three receptor residues: Lys197^2.67b^ forms a stable contact with Phe10 of TIP39, Arg305^ECL2^ establishes a salt bridge with Glu21^P^, and Tyr318^5.39b^ engages Asp7^P^ via a hydrogen bond. Furthermore, the unique cyclic N terminus of TIP39 contributes predominantly (65%) to the binding interface, stabilizing it between TM5 and TM6 of PTH2R (20). Critically, a single residue substitution (L370^6.47b^P) in TM6 enables PCO371, a small-molecular PTH1R agonist, to activate PTH2R (21), informing the rational redesign of existing small-molecule drugs for PTH2R specificity.

Mature human and previously purified bovine peptide sequences for TIP39 are identical. Likewise, it is predicted that mouse TIP39 is identical to rat TIP39. The amino acid sequence is highly conserved, differing only in positions 24, 27, 31, and 35. These imply that TIP39 peptides may have a conserved function across different species (22).

### 3.2 Anabolism

TIP39 is encoded by human chromosome 19q13.33 and is processed from a preprohormone. The transcription and translation of TIP39 are systematically described in Homo sapiens and Mus musculus (2, 23). The preprohormone consists of 100 aa (amino acids), which is then sequentially processed into a 70 aa prohormone and a final 39 aa neuropeptide. The TIP39 preprohormone is encoded by two exons; one encoding 43 aa of the leader sequences, and the other one encoding 57 aa. The initial 30 amino acids are likely involved in signal peptide functions. Additionally, two potential cleavage sites are predicted, both featuring an Arg–Arg structural motif. One is found between the 31-residue internal peptide and the 39-residue excreted peptide, while the other is at the 22/23 position within the 39 aa sequences. However, the function of the second site is uncertain. It could contribute to in the processing, breakdown, or inactivation of TIP39, or the cleavage products might possess biological activity. In addition, there’s no indication of posttranslational modifications in any of the amino acids (4).

### 3.3 Receptor: PTH2R

During the investigation of GPCRs within the central nervous system, PTH2R, a type B1 GPCR, was discovered. It exhibits 51% amino acid sequence similarity to PTH1R, a receptor from the same family. Furthermore, it has been established that PTH can trigger its activation before finding TIP39 (15, 24). However, PTH mRNA couldn’t be detected in the brain where PTH2R mRNA is abundant (14).

Experimental data concerning anatomical connections, receptor selectivity, and ligand specificity suggest that TIP39, rather than PTH, is considered the natural ligand for PTH2R. The patterns of TIP39-positive fibers and PTH2R-expressing neurons and their projections are remarkably similar across the brain. Although there are slight variations between different species, generally, TIP39 selectively binds and activates PTH2R, with no discernible effect on PTH1R (25). The interaction strengths of PTH2R with the three key functional peptides: potent (TIP39), moderate (PTH), and weak (PTHrP). (7-39) TIP39 is an effective but nonselective antagonist of PTH2R (26). Histidine^4^, tyrosine^5^, tryptophan^6^, and histidine^7^-TIP39 (HYWH-TIP39) have been developed as high-affinity antagonists for PTH2R (27).

#### 3.3.1 The distribution of PTH2R

The localization of PTH2R has been exclusively investigated in mice, rats, macaques and humans (5, 25, 28, 29). Unlike the more centralized distribution of TIP39-expressing neurons, neurons expressing PTH2R were widely distributed. PTH2R expresses both in peripheral and nervous systems.

##### 3.3.1.1 In the peripheral tissue

Using antibody labelling techniques, PTH2R expression was detected in a series of rat endocrine cells, including pancreatic D cells, thyroid parafollicular cells, and certain gastrin-producing cells. PTH2R mRNA expression was 54-fold upregulated in the islets of obese patients (30). It is suggested that the peripheral TIP39-PTH2R system may participate in regulating pancreatic function and calcium ion homeostasis within the body (7, 24).

PTH2R expression was also detected in bone and tracheal cartilage cells of rats by using antibody labelling techniques, indicating their involvement in skeletal system development (29). In a cross-sectional population study, lower levels of TIP39 and PTH2R transcription were detected in whole blood samples from women with osteoarthritis compared to the general population (31). Interestingly, variations in the PTH2R gene correlate with lumbar degenerative changes in postmenopausal women (32). Furthermore, the expression level of the PTH2R gene is upregulated in patients with breast cancer bone metastases (33). What’s more, TIP39 inhibits the proliferation and differentiation of chondrocytes (34), and disruption of the PTH2R is closely associated with non-syndromic craniosynostosis (35).

PTH2R has been shown to be expressed in endothelial cells and smooth muscle cells of the heart and aorta of rats by using *in situ* hybridization (ISH). *In-vivo* experiments indicated that TIP39-PTH2R lacks vasodilatory effects but exerts potent negative inotropic action (36). In the ischemia-reperfusion model, exogenously administered TIP39 effectively reduces coronary vascular resistance (37). Furthermore, *in-vitro* experiments in rats indicated that PTH2R expressed in the renal artery exerts a vasodilatory effect (38). In human diabetic nephropathy, upregulation of genes, including PTH2R, in protective macrophages is linked to their depletion, podocyte injury, and apoptosis (39).

ISH has detected PTH2R mRNA expression in mouse and human testes, and it is essential for germ cell maturation. Knockout TIP39 male mice are sterile (40). Furthermore, immunoblotting analysis confirms the presence of TIP39 in buffalo seminal plasma (41).

Research had revealed that keratinocytes and adipocytes in both human and mouse skin also express PTH2R by using RT-PCR, immunohistochemistry, and Western blot, and exogenous TIP39 slows the growth of keratinocytes and induces their differentiation (42). Furthermore, TIP39 can promote extracellular matrix formation and wound healing (43).

PTH2R mRNA expression was significantly elevated in CD34+ MDS and CD34+ CD38- AML cells compared to normal cells, correlating with poorer prognosis. *In-vitro* experiments demonstrated that TIP39 induces autophagy in leukemia cells by inhibiting mTOR, thereby reducing leukemia cell apoptosis (44).

Clinical data analysis indicates that the PTH2R gene is highly expressed in ovarian cancer cells, and it participates in the proliferation, invasion, and metastasis of ovarian cancer cells (45).

Unlike rat lung tissue, previous northern blot analyses of human PTH2R mRNA failed to demonstrate its expression in lung tissue (24). Interestingly, a case report on lung adenocarcinoma revealed novel PTH2R-ALK fusion mutations (46). Furthermore, single nucleotide polymorphism analysis indicated an association between PTH2R and asbestos-induced lung cancer (47).

In summary, PTH2R is distributed throughout the pancreas, skeletal system, cardiovascular system, reproductive system, and skin, both in rats and humans. Furthermore, PTH2R is also present in the rat thyroid, gastrointestinal tract, and certain human tumor cells. It likely plays a crucial regulatory role in pancreatic islet function, calcium homeostasis, skeletal growth and development, skeletal diseases, cardiovascular disorders, reproductive function, skin keratinization, and tumor initiation and progression.

##### 3.3.1.2 In the mouse brain

The distribution of PTH2R-expressing cell bodies detected by ISH and by labeling beta-galactosidase driven by the PTH2R promoter in knock-in mice was almost identical.

Cerebral cortex: Neuron density was highest across the infralimbic cortex (IL), with especially dense populations in specific frontal areas [including the prelimbic (PrL), anterior cingulate (ACC), and insular cortices (Ins)] and within layer 6b located on the outer boundary of the corpus callosum (cc). Moving caudally, neuron density progressively decreased, although it remained elevated in the uppermost regions of the ectorhinal cortex (Ect).

Limbic system: PTH2R-expressing neurons were sparsely found in the anterior olfactory nucleus (AON) and the olfactory tubercle (Tu). These neurons were found in high concentrations in the ventral and medial parts of the lateral septal nucleus (LS). The bed nucleus of the stria terminalis (BST) showed moderate density overall, with the posterior medial region having particularly high density. The medial nucleus of the amygdala had a substantial number of PTH2R-positive neurons, while moderate densities were found in the anterior amygdaloid area (AA), central and cortical amygdaloid nuclei (Ce,Co), and amygdala-hippocampal transitional zone (AHi). The neurons were sparsely distributed throughout the hippocampus (HIP), with somewhat elevated levels in the dentate gyrus (DG) and subiculum (S).

The basal ganglia: The neurons with PTH2R expression are prominently located in the claustrum (Cl) and dorsal endopiriform nucleus (DEn), while their presence is comparatively sparse in the accumbens nucleus (Acb), caudate putamen (CPu), ventral striatum (STRv), and substantia innominate (SI).

Thalamus: The medial subregion of the medial geniculate body (MGM) in the posterior thalamus exhibited a dense population of PTH2R-positive neurons, while moderate densities were observed in the paraventricular (PV), centrolateral (CL), paracentral (PC), centromedian (CMn), and reuniens thalamic nuclei (Re), along with the lateral habenular nucleus (LHb) and the subthalamic zona incerta (ZI). In contrast, other thalamic regions displayed relatively fewer neurons.

Hypothalamus: The presence of a considerable concentration of PTH2R-labeled neurons was discerned within neuroendocrine regions, including the preoptic area (POA) and the periventricular (Pe), paraventricular (PVN), and arcuate nuclei (Arc) (48). Substantial neurons in POA suggest that the TIP39-PTH2R system is essential for rodent maternal behavior (18). In addition, the medial and lateral subdivisions of the superior mamillary nucleus (MM, LM), the ventral premamillary (PMV), and the tuberomammillary nuclei (TM) also contained many neurons.

**Figure 2:**
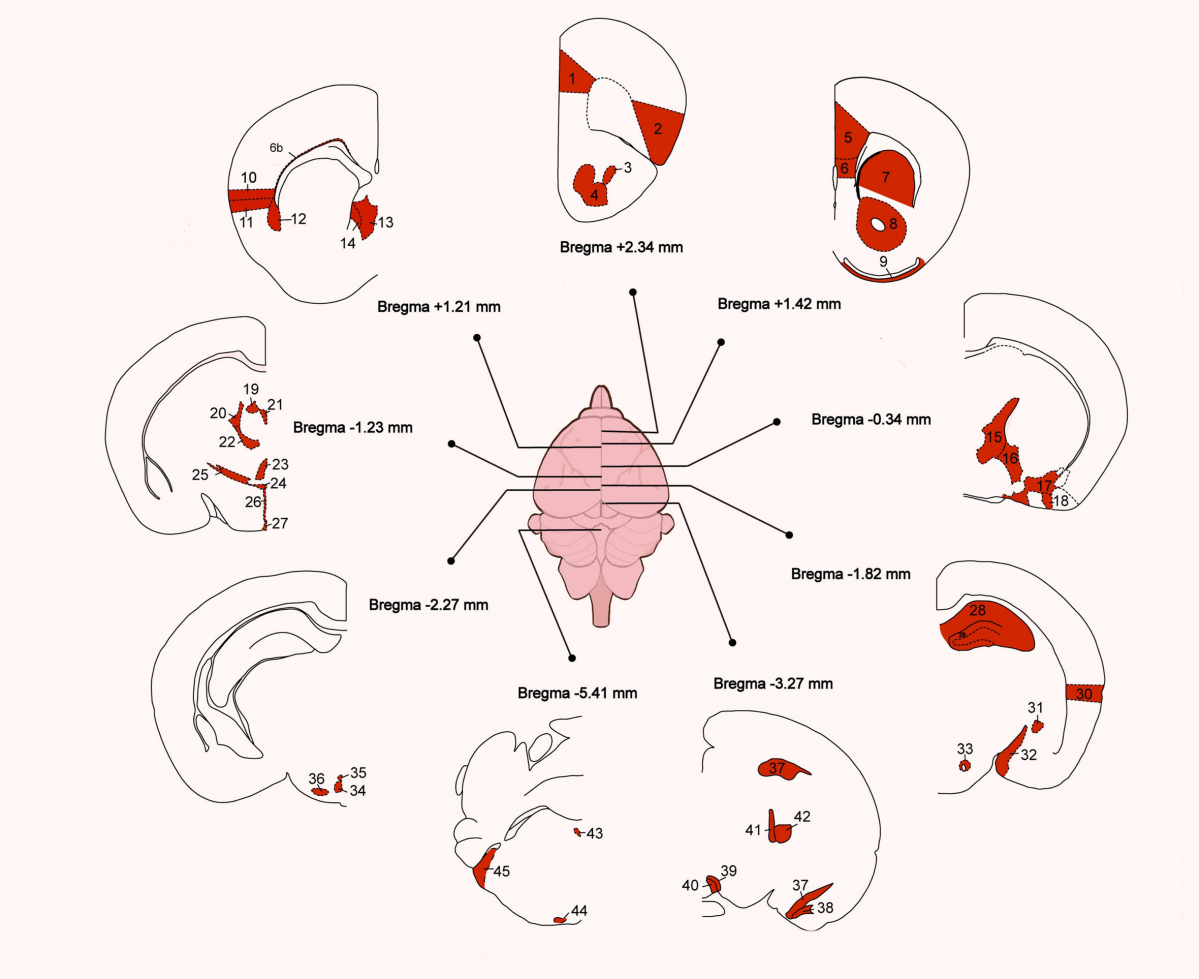
Distribution of PTH2R-expressing neurons in the mouse brain (49).

In addition, neurons that express PTH2R are found in lower numbers in the brainstem and cerebellum, yet exhibit a relatively higher density in specific regions, including the lateral interpeduncular nuclei (IPL), the paranigral nuclei (PN), the medial raphe nucleus (MR), the sphenoid nucleus (Sph), the nucleus of the trapezoid body (Tz), the cochlear nuclei (CN), and the nucleus of the solitary tract (Sol). A concise overview of the distribution of PTH2R is provided in the following schematic, adapted from the work of Faber, Catherine A et al. (**Figure 2**) (25).

1. PrL, prelimbic cortex; 2. AI, agranular insular cortex; 3. DEn, dorsal endopiriform nucleus; 4. AON, anterior olfactory nucleus; 5. Cg, cingulate cortex; 6. IL, infralimbic cortex; 7. CPu, caudate putamen; 8. Acb, accumbens nucleus; 9. Tu, olfactory tubercle; 10. GI, granular insular cortex; 11. DI, dysgranular insular cortex; 12. Cl, claustrum; 13. LSI, lateral septal nucleus, intermediate part; 14. LSV, lateral septal nucleus, ventral part; 15. BST, Bed nucleus of the stria terminalis; 16. SI, substantia innominate; 17. AA, anterior amygdaloid area; 18. Co, cortical amygdaloid nucleus; 19. LHb, lateral habenular nucleus; 20. CL, centrolateral thalamic nucleus; 21. PV, paraventricular thalamic nucleus; 22. PC, paracentral thalamic nucleus; 23. Re, reuniens thalamic nucleus; 24. PVN, paraventricular nucleus (PVN); 25. ZI, zona incerta; 26. Pe, periventricular hypothalamic nucleus; 27.Arc, arcuate hypothalamic nucleus; 28. HIP, hippocampus; 29. DG, dentate gyrus; 30. Ect, entorhinal cortex; 31. Ce, central amygdaloid nucleus; 32. Me, medial amygdaloid nucleus; 33. PeF, perifornical nucleus; 34. DM, dorsomedial hypothalamic nucleus; 35. DTM, dorsal tuberomamillary nucleus; 36. PMV, premamillary nucleus, ventral part; 37. S, subiculum; 38. AHi, amygdalohippocampal area; 39. PN, paranigral nucleus of the ventral tegmental area; 40. IPL, interpeduncular nucleus, lateral subnucleus; 41. MGM, medial geniculate nucleus, medial part; 42. MGV, medial geniculate nucleus, ventral part; 43. Sph, sphenoid nucleus; 44. Tz, nucleus of the trapezoid body; 45. VCN, ventral cochlear nucleus. The diagrams are modifications from the Allen Mouse Brain Atlas.

##### 3.3.1.3 In the rat, macaque, and human brain

Using both immunohistochemistry and ISH, the distribution of PTH2R-expressing cell bodies in rats closely mirrors that observed in mice, with notable exceptions (50). Specifically, in the medial septal (MS), ventromedial hypothalamic (VMH), parabrachial (PBN), spinal trigeminal nuclei (Sp), and periventricular (Pe) regions, the neuronal density is augmented in rats relative to mice. Furthermore, PTH2R expression has been exclusively documented in the spinal cord and sensory ganglia of rats. Additionally, while PTH2R-expressing neurons are abundant in the posteromedial part of the medial division of the bed nucleus of the stria terminalis (STMPM), the medial geniculate body (MG), and the nucleus of the trapezoid body (Tz) in mice, these areas exhibit minimal expression in rats (25). ISH in macaque tissue and RT-PCR in human tissue revealed that the cerebral distribution of PTH2R is highly like that observed in rodents (7).

The regions of TIP39- and PTH2R-containing fibers extensively intersected with those of PTH2R-expressing neurons. However, in certain regions, fibers containing PTH2R were detected without TIP39-containing fibers. The ventral part of the lateral geniculate nucleus (LG), the dorsomedial and central subdivisions of the VMH, the dorsal subdivision of the interpeduncular nucleus (IPD), and the cerebellum are included (25). Despite the aforementioned observations, the overlap of PTH2R and TIP39 fibers suggests that the TIP39-PTH2R system may function through an axo-axonic mechanism. Additionally, PTH2R and VGLUT-2 showed co-expression at synaptic terminals within the septum (SPT) and hypothalamus, while glutamic acid decarboxylase was absent (51), suggesting that neurons that express PTH2R are probably glutamatergic but not GABAergic (7).

#### 3.3.2 The TIP39-PTH2R interaction

The TIP39-PTH2R interaction model is like the two-site model, which is hypothesized to mediate the PTH/PTHrP-PTHR interaction. The engagement of the ligand’s C-terminal region with the receptor’s extracellular N-terminal domain (N interaction) is essential for the initial binding, while the interaction of the ligand’s N-terminal moiety with the receptor’s juxtamembrane domain (J interaction) drives receptor activation (15). Experiments involving peptide truncation, residue substitution or modification, and chimeric receptor construction provide support for the proposed interaction model (3, 17).

However, there are certain distinctions in the interaction mode between TIP39 and the PTH2 receptor. The J interaction is also pivotal in governing the selectivity of binding, whereas specific charged residues located on the C terminal of TIP39 may attenuate the contribution of the N interaction on the overall binding strength (3, 16). Furthermore, more residues located at the C terminus of TIP39 contribute to its activity (22). In addition, compared to the uncoupled state, the coupling of the G protein with the receptor amplifies the binding specificity of PTHrP for PTH1R and TIP39 for PTH2R (3, 15).

#### 3.3.3 The activation effect

TIP39 has been demonstrated to elevate cAMP levels and enhance intracellular Ca ion concentrations by mobilizing calcium from intracellular stores through Gs and Gq signaling pathways, respectively (52). The two pathways, adenylate cyclase and PLC/calcium, differ in their dependence on the number of TIP39 N- and C-terminal residues. A truncation of 6 aa from the N terminus completely eliminates the activity of the cAMP pathway, whereas in the PLC/calcium pathway no elevation of calcium ions could be detected by truncating only four peptides. Likewise, TIP39(1-30), after a 9-amino acid truncation at the N-terminus, retains its ability to fully activate the cAMP pathway but only partially activates the PLC/calcium signaling pathway (22). However, it is not clear whether both or either occur in different cell populations expressing PTH2R (52).

In addition, DAG and reduced levels of PI (4,5) P2 are directly involved in TRPC (transient receptor potential canonical) channel activation, which is a nonselective cation channel through which calcium ions can pass (53). Yet another observation of increased expression of calcium-activated chloride channel genes after TIP39 action (53), as well as the fact that anoctamin-1, a calcium-activated chloride channel, is complexed with some G-protein coupled receptors, and then the plasma membrane component of this complex binds to the ER through the interaction of anoctamin-1 with IP3R1, suggests that TIP39-PTH2R may regulate intracellular calcium and chloride levels through the interaction of GPCR with ion channels (54).

During the post-activation phase, the receptor typically recruits β-arrestins, which are instrumental in regulating the cAMP response pathway and also contribute to receptor internalization and desensitization. However, overexpression of dominant negative forms of either β-arrestin or dynamin only minimally affected internalization of PTH2R. It is suggested that PTH2R may also facilitate receptor internalization and desensitization via a β-arrestin-independent pathway involving PKCβ. Whereas the key site of PTH2R that interacts with β-arrestins is the region between residues 426 and 457 at the C-terminus, and the intracellular loop 3 (IC3) domain of PTH2R stabilizes receptor-inhibitory protein interactions, which is also important for receptor-Gs coupling. However, it is important to emphasize that the normal response of PTH2R to TIP39 is characterized by internalization together with β-arrestins. One conjecture is that this complex may possess other biological functions yet to be investigated (55).

As current research indicates, the theory of biased GPCR signaling proposes that biased agonists can selectively activate either G protein or β-arrestin pathways, thereby enhancing therapeutic efficacy while reducing side effects (56). Given the *in vivo* observations that PTH2R internalization and desensitization appear to occur independently of β-arrestins (55), the development of biased agonists for PTH2R holds promise not only for therapeutic applications but also for elucidating the potential unique role of the β-arrestin pathway in PTH2R signaling.

#### 3.3.4 Blood–brain barrier penetration and feasible central delivery routes

TIP39 has a molecular mass of 4504.5 Da as determined by mass spectrometry (4), and its cryo-EM structure reveals an amphipathic nature (X. 20). Currently, research on the central effect of the TIP39-PTH2R system remains at the preclinical stage, with administration routes restricted to intracerebral or intrathecal injection; the delivery strategy involved is exclusively lentiviral vector delivery (57). As a result, there is no evidence to suggest that TIP39 can cross the blood–brain barrier.

Advances in neuropeptide delivery may provide a framework for the central delivery of TIP39. Intranasal drug delivery has emerged as a highly promising route for neuropeptide administration to the brain. This method enables drug transport to the brain via both the olfactory and trigeminal nerves (58). For instance, neuropeptide Y has been administered intranasally for the treatment of major depressive disorder in humans (59).

Furthermore, various forms of nanoparticles have been demonstrated to effectively deliver pharmaceuticals. Liposomes are considered one of the most extensively researched and clinically validated peptide delivery nanocarriers, owing to their superior stability and reduced encapsulated drug toxicity. They also permit surface modification with targeting ligands to achieve site-specific delivery (60). In addition, the conjugation of cell-penetrating peptides (CCP) represents an effective intracellular delivery strategy, enabling the co-transportation of both the peptide and its drug payload into cells via direct translocation or endocytosis (61), and the approach has been successfully employed in rodent models of depression (62).

Mesenchymal stem cell-derived extracellular vesicles (MSC-EVs) represent a promising vehicle for protein delivery (63). What’s more, focused ultrasound-mediated microbubble cavitation temporarily opens the blood–brain barrier, serving as an efficient means for delivering bioactive materials into the brain (64).

Overall, there is currently no evidence to suggest that TIP39 can cross the blood–brain barrier, and further research is required to develop strategies for delivering the peptide into the brain.

## 4 Expression and distribution of TIP39 in brain

Based on non-quantitative RT-PCR, whole-mount ISH and northern blots, TIP39 is detectable in the testis, the eye, the dorsal root ganglia, kidney, pancreas, trachea, liver, and several regions of the brain. Of them, TIP39-containing neurons locate relatively more restricted in the brain compared with that in other organs (5, 23, 36, 38, 65–67) (As described in **Table 1**).

**Table 1:** Distribution of TIP39 in organs.

Organ	Species	Method	Possible effects	Reference
Brain (thalamus, amygdala, locus coeruleus, hypothalamus)	human	RT-PCR	Endocrine effects. Modulation of auditory response, nociception, fear response. Anti-depressive effects. Modulation of maternal behavior.	(2)
	macaque	ISH		(4, 5
	rat	RT-PCR		(65)
	mouse	ISH		(23)
	tilapia	rQRT-PCR		(67)
	zebrafish	whole-mount ISH		(66)
DRG	rat	RT-PCR	Modulation of nociception.	(65)
spinal cord	mouse	double-label IHC		(65)
trachea	human	RT-PCR	–	(2)
liver	human	RT-PCR	–	(2)
	mouse	Northern blot		(23)
kidney	human	RT-PCR	–	(2)
	rat	RT-PCR		(38)
	mouse	Northern blot		(23)
	tilapia	rQRT-PCR		(67)
heart	human	RT-PCR	Negative inotropic effect?	(2)
	rat	RT-PCR		(36)
	tilapia	rQRT-PCR		(67)
	zebrafish	whole-mount ISH		(66)
eye	rat	RT-PCR	–	(65)
testis	rat	RT-PCR	Spermatogenesis and ejaculation.	(65)
	mouse	Northern blot, ISH		(23)
	tilapia	rQRT-PCR		(67)

PT-PCR, reverse transcription polymerase chain reaction; ISH, *in-situ* hybridization; rQRT-PCR, real-time quantitative RT-PCR; DRG, the dorsal root ganglia; IHC, immunohistochemistry

Different brain regions correspond to different functions and work in complex, intertwined ways. To dive into functions of TIP39, knowledge about its distribution cannot be ignored. In macaque and human brains, TIP39 locates similar to that in rodents (1, 5). It is originally synthesized in the thalamic subparafascicular area (SPF) and the caudal paralemniscal nucleus, then projects to PTH2R in the brain (5, 65, 68).

### 4.1 TIP39 expression in rat’s brain

Focusing on the rodents’ brain, TIP39 is highly expressed in three regions of the lateral pons and the SPF in the caudal thalamus: the MPL, the PVG, and the PIL (5, 7). Of them, periventricular thalamic neurons constitute the most substantial amount of TIP39 cells in young adult rodents’ brains (25, 69), with about 600–1000 neurons per side. About 75% of the MPL cell population is made up of TIP39-express neurons (68). The spatial distribution of TIP39 in the brain changes over time (70) and shows a notable sexual dimorphism in adult rodents’ brains (71).

#### 4.1.1 TIP39 expression in a rat’s developing brain

In the embryonic and neonatal rats’ brains, TIP39 is mainly confined to the SPF, with a few cells extending into the PIL and the MPL nearby. Dávid Brenner found that TIP39 expression in immature rat brains first displays at embryonic day 14.5 (ED-14.5) in the MPL, while that in the SPF appears at postnatal day 1 (PND-1) (70). Overall, the level of TIP39 mRNA climbs to the maximum at postnatal day 14 (PND-14) (71); then the level of TIP39-immunoreactive (TIP39-ir) neurons peaks by postnatal day 33 (PND-33) and almost completely disappears by postnatal day 125 (PND-125). It later reappears in mature brain (70). Yet rigorous to say, the data of temporary disappearance of detectable TIP-ir cell bodies provides no evidence whether TIP39 neurons degenerate or whether their TIP39 levels just decrease below the minimum detection threshold during embryonic development (71).

#### 4.1.2 TIP39 expression in adult rat’s brain

When it comes to mature rats, the general distribution of TIP39 cell bodies is similar to that in developing rat brains. ISH and immunocytochemistry reveal that, still, they are confined in the SPF and the MPL, mainly concentrated in the MPL at the ponto-mesencephalic junction, with few bodies scattered in the dorsal and dorsolateral hypothalamic areas (72).

##### 4.1.2.1 TIP39 in the PVG

Injections of the anterograde tracer biotinylated dextran amine and the retrograde tracer cholera toxin B subunit have clearly mapped that TIP39 neuron bodies’ distribution in the PVG goes diagonally backward in a sigmoid shape, corresponding to the shape of the brain region (7, 23, 73). Most of the neurons are situated medial to the magnocellular subparafascicular nucleus (MSPF); some lie ventral to the central thalamic nucleus (CM), dorsal to the posterior hypothalamic nucleus (PHA), and medial to the parvicellular ventral posterior nucleus of the thalamus (VPMpc) (25, 72). When it comes to caudal TIP39 neurons, more of the cell bodies are located dorsally between the midline and the fasciculus retroflexus (FRF) (7); a few are aligned near the caudal end of the third ventricle (TV) (23, 73); others appear more laterally, situated ventrally to the FRF (23, 73). The range of TIP39 neurons persists up to the level of the posterior commissure, followed by a plunge in neuronal density (7).

##### 4.1.2.2 TIP39 in the PIL

The posterior intralaminar complex of the thalamus, or the PIL for short, is a caudolaterally elongated topographical brain area (74). ISH shows that in the PIL, TIP39 cell groups are visible per side between bregma levels −4.2 to −6.1 mm. Compared with that during embryonic and early postnatal development, the TIP39 number in the PIL is substantially lower, only about 200–300 neurons (70, 73). The laterally situated TIP39 neurons arrange themselves into a horizontal cellular row within the parvicellular subparafascicular nucleus (SPFp), an anatomical region overlying the medial lemniscus. From this origin, these neurons continue their lateral alignment, extending their reach towards the posterior intralaminar thalamic nucleus (PITN), nestled at the ventromedial border of the medial geniculate nucleus (MGN) (25, 73). Furthermore, a subset of TIP39 neurons, spanning bregma levels between −4.5 and 5.2 mm, is distributed beneath the lateral aspect of the medial lemniscus (ML), residing within the lateral confines of the caudal zona incerta (CZI) (25, 71).

The rostromedial-to-caudolateral arrangement of TIP39 neurons exhibits a parallel orientation with the distribution of calcitonin gene-related peptide (CGRP)–containing neurons within the posterior thalamus. The PAP double labeling method indicates that these two kinds of neurons do not overlap despite being in the lateral part of the posterior thalamus (PoT) (71). In coronal sections, the majority of TIP39 neurons are positioned medially relative to the bulk of CGRP neurons (70). The result of c-fos suggests that TIP39 neurons in the SPFp are in the medial subdivision of the area (75). Furthermore, TIP39 and CGRP neurons extend and maintain their medio-lateral separation even after leaving the SPFp (75) and display diverse projection patterns (76–78). These constitute evidence that not only the SPFp but the whole PIL can be subdivided into medial segments populated by TIP39 neurons and lateral segments inhabited by CGRP neurons (7).

##### 4.1.2.3 TIP39 in the MPL

The MPL, initially introduced to delineate the location of TIP39 neurons (72), has been incorporated into the Paxinos rat brain atlas since 2005.

The MPL contains TIP39 neurons (300–600 per side), glial cells (which account for about one-third of all cells), a small group of large acetylcholinesterase-positive cells of the epirubrospinal nucleus (ERBN), and the A7 noradrenaline cell group medially. Distinguished from those in adjacent areas, cells in the MPL are separated into dorsolaterally oriented cell columns by 20- to 50-μm-wide cell-free zones. The four kinds of cells distributed are superimposed on each other. However, the functional significance of the spatial relationship between TIP39 neurons and noradrenaline neurons remains an area ripe for further exploration (68).

According to fluorescent Nissl staining combined with immunolabeling in rat brain, TIP39-containing cell bodies are situated in the rostral pons between bregma levels −8.0 and 8.5 mm (23, 25, 65), medial to the fibers of the lateral lemniscus, immediately dorsal to the rubrospinal tract, and rostral to the Kölliker-Fuse nucleus (68).

#### 4.1.3 Sexual dimorphism of adult TIP39 expression

Additionally, compared to convergent TIP39 expression in immature brains (25, 71), there rises a novel characteristic when brains have fully developed, defined as sexually dimorphic (71). Gender-dependent difference occurs notably in the brain of aged rats. By means of ISH, quantitative RT-PCR, and immunocytochemistry, TIP39 levels markedly decreased as compared to younger animals (25, 72), yet those in female brains are significantly higher than those in male brains. But if male rats are castrated before puberty, the gender gap in TIP39 levels can be slightly edged up (71).

It is hypothesized that the emergence of TIP39 during early postnatal development, followed by its decline later, along with gender-specific expression levels in mature animals, hints at a possible involvement in sexual maturation or gender-specific physiological processes (79).

### 4.2 TIP39 projection in rat’s brain

Various techniques have been applied to clarify the projection models of the three groups of TIP39 neurons. As early as the third year after the discovery of TIP39, 2000, anterograde tracers were reported to be injected into the PIL (80). This method was also used to establish projection patterns in the PVG (73). To move on, the PVG, the PIL, and the MPL were lesioned, respectively, and the disappearance of TIP39-containing fibers in different brain regions was analyzed then (69). A combination of targeted bi- or unilateral lesions brought a great amount of information on the projection of each TIP39 cell groups individually (69). In addition, retrograde tracers into TIP39 fiber terminals in rich encephalic regions reveal the origin of projections, bringing patterns of multicellular population projections and bilateral projections to light (73).

**Figure 3:**
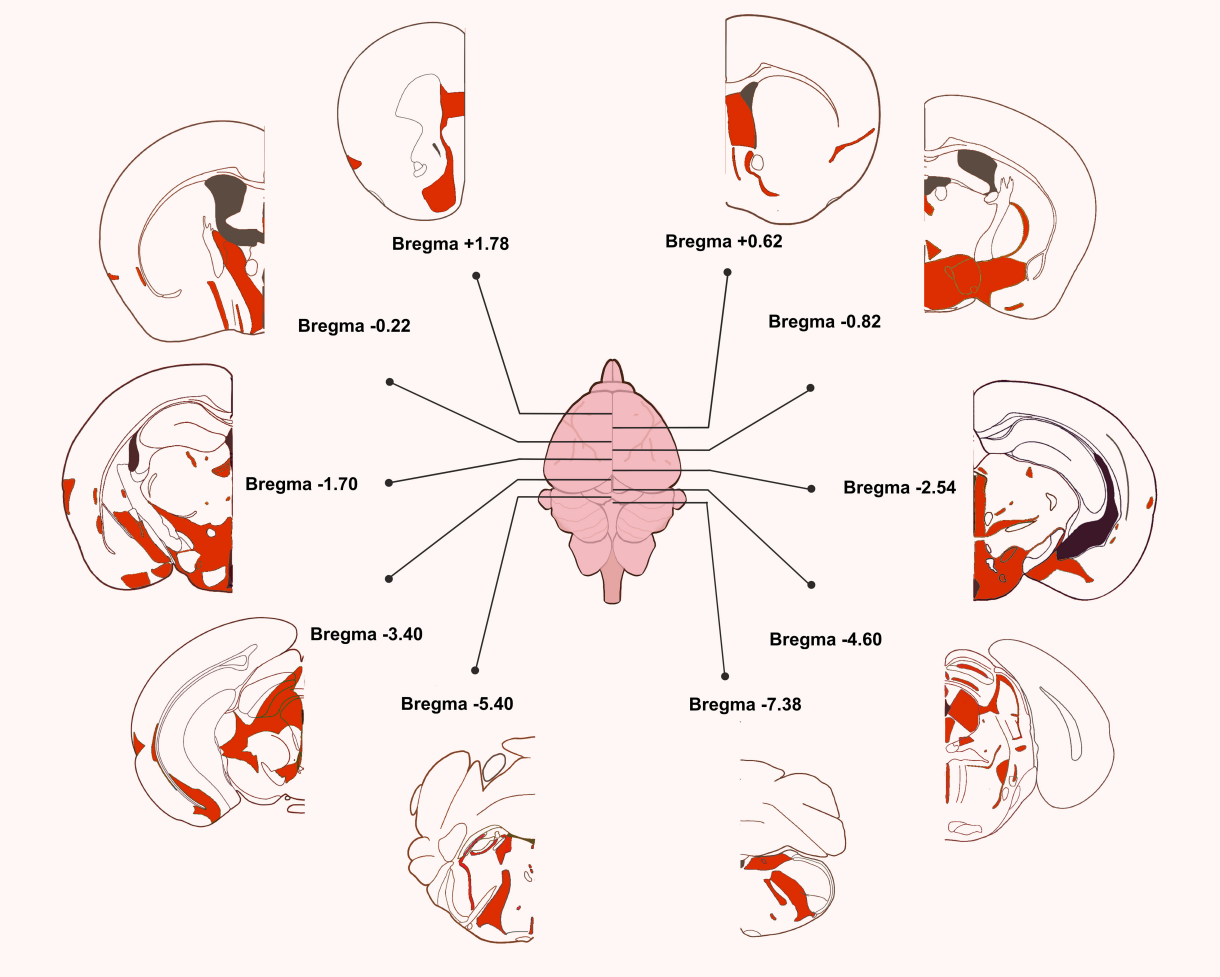
Schematic diagrams demonstrate the distribution of TIP39 in the brain of mice (49).

In contrast with the restricted localization of TIP39-expressing cell bodies, TIP39-containing fibers are much more extensively distributed (52, 72). The emitted projections are highly co-localized with PTH2R (7) (**Figures 2**, **3**).

The diagrams are modifications from the Allen Mouse Brain Atlas (CC BY 4.0), all scaled and displayed on the basis of the original size of each brain section.

By and large, TIP39 neurons in the rostral and caudal part of the SPF widely spread to the cerebral cortex. Cortex regions that receive TIP39 fibers mainly include the medial prefrontal (mPFC), insular (Isl), ecto-and-perirhinal cortex (EPC) (72). In addition, a great number of fibers reach nucleus of the diagonal band (MDSV), septum, central and basomedial amygdaloid (Amyg) nuclei, fundus striati (FS), basal forebrain (BF), midline and intralaminar thalamic nuclei (MITN), hypothalamus, subthalamus (STh) and the periaqueductal gray (PAG) (73).

#### 4.2.1 TIP39 in the PVG

Double-labeled neurons demonstrate an even distribution of TIP39 in the PVG, indicating that TIP39 neurons in this region form a distinct cell population (73). According to results of electrolytic lesions (69) and anterograde tracer injection (using biotinylated dextan amine; BDA) (73), TIP39 neuron mass in the PVG projects to the mPFC, the shell and cone portions of the nucleus accumbens (SNAC), the lateral septum (LS), the bed nucleus of the stria terminalis (BNST), the Amyg, the FS, the ventral subiculum (vSub), the thalamic PVN, the hypothalamus, and the PAG (69, 73). Projections from this area are dominantly ipsilateral (69).

By tracing anterogradely labeled fibers, the projection pathway of TIP39 can be mapped. TIP39 projections from the PVG primarily follow two sets of pathways.

One group of fibers exits the PVG dorsally, makes a hook-shaped turn into the epithalamus (EP), and then runs rostrally within the PVN. Some scattered fibers also run rostrally from the PVG dorsal to the medial lemniscus, likely terminating in thalamic nuclei (73). These neurons are expected to play roles in nociceptive effects, which is previously demonstrated (65).

A second, larger group of TIP39 fibers connects the zona incerta (ZI) and the supraoptic decussations (SOD), entering the basal forebrain area (BFA). From there, the fibers branch out to limbic cortical, septal, and amygdaloid regions (73).

The PVG is the most important TIP39-related brain region. It is the PVG, but not other TIP39-containing regions, that is labeled TIP39 perikarya. Accordingly, it is speculated that the PVG provides the original and only TIP39 input to the PIL and the MPL (73).

Notably, after PVG-BDA injection, some hypothalamic areas, including the anterior, paraventricular, arcuate, and dorsomedial nuclei, show a significantly higher density of TIP39-ir than TIP39-BDA double-labeled fibers (73). This data suggests that the PVG contributes only minimally to TIP39 fibers in most hypothalamic areas.

#### 4.2.2 TIP39 in the PIL

Anterograde tracer injection (80) and lesions of the PIL and surrounding areas (69) reveal the routing and termination points of TIP39-laden fibers. TIP39 fibers project to the ipsilateral amygdala. A sharp plunge of TIP39 levels was observed in the ipsilateral hypothalamus after PIL injury, most prominently in the paraventricular and dorsomedial nuclei. In addition, moderate yet visible reductions happened in forebrain regions, including the mPFC, the nucleus accumbens (NAc), and the BNST (69).

Further studies utilizing the anterograde tracer method have provided additional support for the widespread neuronal projections in the rat brain (80). The results corroborate previous studies on major projections of TIP39 from the PIL to the amygdaloid complex and the ventromedial nucleus of the hypothalamus (VMH) (74, 80). Fibers are concurrently found in the temporal cortex (TC) and medial preoptic area (MPOA) (74, 81). Subsequent retrograde studies further identified TIP39 projections originating from the PIL and terminating in the PVN (80, 82). Additionally, it is hypothesized that TIP39 neurons within the PIL may also serve as the origin of TIP39 fibers in the ectorhinal cortex (ECT), the PAG, the external cortex of the inferior colliculus (IC), and the periolivary area (7).

### 4.2.3 TIP39 in the MPL

Following lesion of the MPL, TIP39 fibers almost completely disappeared from the deep layers of the superior colliculus (SC), the external cortex of the IC, the cuneiform nucleus (CNF), the lateral parabrachial nucleus (LPB), the medial nucleus of the trapezoid body (MNYb), and the periolivary area, yet there is still a moderate density of TIP39-ir fibers remaining in the PAG (69). According to Stefan Reuss, TIP39 neurons in the MPL project to periolivary regions of the superior olivary complex (SCO) as well (83). Using anterograde tracer injection, Yasui’s team revealed projection from the MPL to brainstem regions (84), but they did not specify which neuron casts the fibers. Since the location of TIP39 neurons in the MPL hasn’t been reliably identified, data regarding the projection pathway of fibers from the MPL are still undetermined.

### 4.3 Interconnection of TIP39 in rat’s brain

Injection of retrograde tracer unveiled cell protection from the PIL and the MPL to PVG, yet the marked cells were TIP39 negative (73). Likewise, following injection of retrograde tracer into the MPL, retrogradely labeled cells in both the PVG and the PIL were non-TIP39 cells, albeit situated in close proximity to TIP39 neurons (68). In addition, a high density of retrogradely labeled cells was also presented in the contralateral MPL, which showed a TIP39 negative response (68). However, existing evidence proposes PVG as the original resource of TIP39 of all TIP39-containing brain regions, encompassing the PIL and the MPL (73).

Apart from the interconnection of the three TIP39-expressing brain regions via projections, another significant association is their massive input to some common projection areas, like the hypothalamic (73, 80, 82). These findings suggest that, despite their apparently different projections, TIP39 neurons located in these three brain regions may be involved in some overlapping functions.

### 4.4 Summary

All three TIP39-containing regions, the PVG, the PIL, and the MPL, project to brain areas including the arcuate nucleus (Arc. Nu.), the hypothalamic area, the periolivary area, and the CNF. The amygdaloid and some forebrain regions, including the mPFC, the IL, the PL, the ectorhinal cortex (EC), and the IC, receive TIP39 fibers from the PVG and the PIL. A great number of TIP39 fibers from the PIL and the MPL co-project to the periolivary area, while fibers in the PAG mainly traced back to the PVG and the MPL. Of the two projections mentioned above, the unmentioned brain area barely provides a small input. It’s notable that, to reach the hypothalamus, TIP39-ir fibers from the PIL and the PVG share two common pathways, travelling through the SOD and the ZI (73, 85). Moreover, TIP39 fibers in brainstem regions and the spinal cord predominately come from the MPL. TIP39 projection mentioned above is summarized in **Table 2**.

**Table 2:** Summary of the projections of TIP39 in the brain of mice.

Situated encephalic region	Projection
the PVG	The medial prefrontal cortex. The shell and cone portions of the nucleus accumbens. The lateral septum. The bed nucleus of the stria terminalis. The amygdaloid nuclei. The fundus striati. The ventral subiculum is the thalamic paraventricular nucleus. The midline and intralaminar thalamic nuclei. The hypothalamus. The posterior intralaminar complex of the thalamus. The term medial paralemniscal nucleus. The periaqueductal gray. The medial lemniscus and terminate. The zona incerta. The supraoptic decussations, Limbic cortical septal. The amygdaloid complex.
the PIL	The amygdala. The hypothalamus. The medial prefrontal cortex. The nucleus accumbens. The stria terminalis. The amygdaloid complex. Temporal cortex, preoptic area. The ventral intermediate septal nuclei. The substantia innominata. The periaqueductal gray. The inferior colliculus. The superior olivary complex.
the MPL	The superior colliculus. The external cortex of the inferior colliculus. The cuneiform nucleus. The lateral parabrachial nucleus. The medial nucleus of the trapezoid body. The periolivary area. The periaqueductal gray. The superior olivary complex. Brainstem.

## 5 The function of TIP39 in the central nervous system

A substantial body of evidence detailing the anatomical distribution of the TIP39 peptide-receptor system provides a critical foundation for elucidating its modulatory roles in the central nervous system (CNS). Previous studies indicated that PTH2R is expressed in several brain parts, including the subparafascicular area, the medial paralemniscal nucleus, and the superficial dorsal horn (DH) of the spinal cord (65, 86). It is reasonable to assume that TIP39 significantly impacts neuronal networks involved in functions such as nociception and audition. Recent enthusiasm and advancements in experimental tools have led to a more comprehensive understanding of TIP39’s physiological function in the brain, and we will discuss the novel findings hereinafter.

### 5.1 Pain

*In situ* hybridization of mRNA encoding the PTH2R revealed strong and intense labeling in both the SPF and MPL areas. Previous research has validated that MPL is closely related to nociception (87), while SPF projects to DH and the caudal region of the sensory trigeminal nucleus (87–89), and these regions contain the majority of the central terminals of primary afferents, which are crucial for the regulation of nociceptive signals. This evidence suggests that TIP39 is likely involved in modulating nociceptive pathways.

TIP39, synthesized by primary afferent neurons and brainstem neurons, may play a role in modulating spinal cord nociceptive information, supported by the presence of PTH2R in both dorsal root ganglia (DRG) and DH neurons, which project into specific domains relevant to TIP39. The administration of TIP39 and PTH2R antagonists in peripheral and central processes demonstrates the involvement of these receptors in nociceptive circuits (69). Mice exhibit a range of dose-dependent nociceptive responses after intrathecal injection of TIP39, including licking, tail-directed scratching, and biting (69, 90–92).

The physiological effects of TIP39 are further substantiated by experiments involving the injection of TIP39 antibodies (69). In a thermal tail-flick assay, injection of the TIP39 antibody increased latency, while the paw withdrawal experiment showed that the latency increased and pressure sensitivity decreased. Subsequent reinjection experiments demonstrated a decrease in latency, reinforcing the predicted facilitatory function of TIP39 in nociception.

The involvement of TIP39 in nociceptive circuits is supported by a clear mechanism. The verifiable hypothesis is that activation of the PTH2R leads to increased intracellular Ca2+ concentrations and cAMP in a DRG neuron-like cell line (F-11 cells) exhibiting peptide-nociceptor properties (91). Two potential pathways describe how these messenger molecules contribute to nociceptive circuits: one involves cAMP regulating the expression of genes responsible for chronic pain, while the other suggests modulation of local synaptic functions. Elevated cAMP levels in nerve terminals facilitate the release of synaptic vesicles, which ultimately leads to spontaneous action potentials and the release of neurotransmitters related to pain (91).

The physiological role of TIP39 in pain modulation indicates its potential for clinical application. Administering TIP39 antibodies, both peripherally and centrally, could represent a promising treatment avenue, warranting further investigation in clinical settings. Additionally, it raises the question of whether chronic pain can be managed through blockade of the PTH2R.

### 5.2 Fear- and stress-related anxiety

Anxiety disorder is among the most prevalent psychiatric disorders and frequently coexists with other conditions, such as depression and somatic disorders (93). This co-occurrence has led to increased interest in exploring anxiolytic treatments. TIP39 is one promising candidate, which may act as a modulator in regulating anxiety during stressful or pathological states, offering a novel approach to address anxiety related to fear and stress.

This hypothesis is supported by the anatomical distribution of TIP39 in the rodent brain. TIP39-positive fibers and PTH2R are widely distributed and found in high densities in key areas associated with stress, fear, and anxiety, including the hypothalamus, amygdala, LS, BST, and mPFC (25, 72).

Emerging evidence further elucidates TIP39’s role in anxiety regulation. Research has shown that administering TIP39 to the central nervous system leads to a reduction in anxiety-like behaviors, as observed in the elevated plus-maze test (9). Furthermore, Fegley et al. provided compelling findings using TIP39 knockout (TIP39-KO) mice, confirming the absence of extragonadal abnormalities in these subjects. In both the elevated plus-maze and the shock-probe test, TIP39-KO mice exhibited heightened defensive burying behaviors and significantly increased anxiety-like behaviors compared to their wild-type (WT) counterparts. Additionally, in the Pavlovian fear conditioning paradigm, TIP39-KO mice displayed more pronounced freezing behavior during both the training phase and in tone and context recall, indicating a strong anxiety response (8).

Another crucial aspect deserving attention is the phenomenon of fear incubation, which refers to the gradual increase in fear response to conditioned cues over time, even in the absence of additional stress or cues (94). It is regarded as an ideal animal model for delayed-onset post-traumatic stress disorder (PTSD), a condition marked by the impaired resolution of fear memories and posing significant treatment challenges (94). Recent behavioral studies suggest that TIP39 likely functions as a neuromodulator influencing fear incubation. For instance, two weeks post-trauma, mice lacking TIP39 signaling exhibited amplified fear recall, anxiety, and depression-like behaviors, while normal TIP39 signaling appeared to mitigate the long-term effects of a single traumatic event following an inescapable footshock (95). These findings were assessed through measurements of contextual freezing, a behavioral indicator of fear memory.

The anatomical and behavioral connections between TIP39 and fear- and stress-related anxiety provide insight into specific underlying mechanisms. The medial nucleus of the amygdala (MeA) is hypothesized to play a crucial role in modulating fear and anxiety, as it is a fundamental component of the neural circuitry involved in both innate and learned fear (96). Several studies support that MEA plays an important role in TIP39 fear regulation. For example, Tsuda MC et al. revealed that the absence of PTH2R-expressing neurons resulted in enhanced fear incubation (97).

Accumulating evidence supports an endocrine role for TIP39 in the regulation of affective behaviors. Previous studies have established that genetic and pharmacological interventions targeting neuropeptide systems, including corticotropin-releasing hormone (CRH), substance P, arginine vasopressin (AVP), galanin, neuropeptide Y, and their corresponding receptors, significantly modulate stress-related and anxiety-like behaviors in rodents. Notably, experimental data demonstrate that TIP39 application in hypothalamic explants stimulates the secretion of key neurohormones, including CRH, luteinizing hormone-releasing hormone (LHRH), growth hormone-releasing hormone (GHRH), and AVP (98). Conversely, intracerebroventricular administration of TIP39 *in-vivo* suppresses AVP release (99). This apparent discrepancy may be attributed to the absence of integrated neural circuitry and physiological feedback mechanisms *in-vitro* preparations. Collectively, these findings suggest that TIP39 contributes to the modulation of anxiety-like behavior through hypothalamic neuroendocrine pathways.

These findings underscore the therapeutic potential of TIP39 in alleviating anxiety and fear; it may offer a novel therapeutic target for anxiety disorders and PTSD.

### 5.3 Cognition and memory

Research has shown that mice lacking the TIP39 signaling pathway exhibited reduced exploration of novel objects compared to their wild-type counterparts (100). Furthermore, these TIP39 knockout mice demonstrated decreased spontaneous alternating behavior in response to arousal conditions induced by novel stimuli, which was accompanied by memory impairments (100, 101). Notably, similar behavioral effects were observed in wild-type mice following acute administration of PTH2R antagonists (100).

The mechanism by which TIP39 may reverse cognitive impairment involves modulation of glutamate and GABA neuronal activity and is also linked to the expression levels of glucocorticoid receptors (GR) and MRs in the brain. Additionally, TIP39 appears to enhance the structure and function of the prefrontal cortex and hippocampus, both of which are crucial regions for cognitive processing in the cerebral cortex.

Previous studies have elucidated the mechanisms underlying cognitive irregularities caused by chronic stress. Chronic stress leads to overactivation of the HPA axis and the PVN, disrupting the balance of glucocorticoid and MRs in the brain (102). This disruption increases excitability at nerve terminals, thus affecting neuroplasticity, excitability, and neuroendocrine regulation at a central level. The process is mediated by GR and MR, which are abundant in the hippocampus and influenced by adrenocorticotropic hormone (ACTH) released from the adrenal cortex. Subsequently, excitatory amino acid neurotransmitters in turn lead to the activation of the HPA axis and an upregulation of GR expression (103, 104).

Pharmacological evidence from studies where TIP39 was injected into the brains of CUMS rats further corroborates its role in addressing cognitive irregularities错误!未找到引用源. The administration of TIP39 substantially reduced the expression ratio of glucocorticoid and MRs in the brain. Moreover, it significantly decreased levels of glutamate and acetylcholinesterase while increasing GABA levels, likely due to enhanced activity of the glutamic acid decarboxylase enzyme. These results highlight the potential of TIP39 in improving cognitive and memory functions.

### 5.4 Social interaction

The significance of social interaction cannot be understated, as it plays a crucial role in stereotypic behaviors related to social recognition and affiliation. Recent studies have highlighted the role of TIP39 in animal social interactions. For instance, in zebrafish, TIP39 expression is induced by the locomotion of nearby fish. Research shows that the transcription of the vertebrate-specific TIP39 decreases following acute isolation but significantly increases when exposed to conspecifics or when receiving information from mechanical disturbances in the water (10). Additionally, TIP39 has been shown to influence social interaction in rodents. Here, we outline two potential pathways through which TIP39 regulates these interactions.

#### 5.4.1 PIL-MPOA pathway

Rich expression of TIP39 has been found in PIL, which is a relay station for processing multi-sensory information (105), while MPOA is also rich in TIP39 terminals. Physical social contact activates neurons in PIL and MPOA (105), and the use of TIP39 and its receptor antagonists has been shown to reduce social grooming behavior, highlighting the role of TIP39 in this pathway.

This newly identified pathway warrants further exploration in the human brain, as the structures of PIL and the distribution of PTH2R in MPOA are similar between rats and humans. Investigating this could potentially lead to novel treatments for conditions characterized by impaired social interaction, such as autism spectrum disorders, independent from the established thalamic-cortical pathways (106).

#### 5.4.2 PIL-PVN pathway

Oxytocin is released from neurons in the PVN and is recognized as a neuromodulator that promotes social behaviors, particularly during non-lactating periods (107, 108). Notably, each oxytocin neuron in PVN is closely connected to TIP39 terminals, and the TIP39 nerve fibers and terminals projecting from PIL activate the oxytocin neurons in PVN. This activation occurs in response to sensory stimuli from the inferior colliculus and the spinal cord following social interactions.

In short, TIP39 plays a crucial role in the excitatory pathway from PIL to PVN, contributing to the release of oxytocin and thereby facilitating social behaviors (109).

### 5.5 Thermoregulation

The median preoptic nucleus (MnPO) serves as the brain’s central hub for thermoregulation (110). It is characterized by a high density of TIP39 terminals and significant expression of PTH2R (25). Consequently, TIP39 is likely to influence thermoregulation.

Evidence supporting this hypothesis emerges from phenotypic changes observed in mice. When TIP39 was administered to the lateral ventricles of wild-type (WT) mice, an increase in core temperature was noted. In contrast, no such increase was observed in PTH2R knockout mice. Additionally, inactivation of the PTH2R gene or acute administration of PTH2R antagonists results in reduced temperature defenses in cold environments (111), while temperature sensation remains normal in PTH2R knockout mice (48), suggesting that TIP39 is involved in heat production rather than temperature sensation itself.

TIP39 may modulate thermoregulation by agonizing PTH2R located in the glutamatergic terminals of the MnPO. Furthermore, projections of PTH2R-containing fibers from the MnPO to the dorsomedial hypothalamus have been confirmed (111). These fibers subsequently extend to the rostral raphe pallidus, ultimately regulating premotor sympathetic output to both brown adipose tissue and cutaneous vascular tone (112, 113).

### 5.6 Endocrine regulation

Regions such as MPOA, ARC, and paraventricular thalamic nucleus (PVT) are ideally situated to influence neuroendocrine functions. Evidence of TIP39’s involvement in endocrine regulation is supported by positive staining with a PTH2R-selective antibody in these areas (29).

Researchers in Bloom’s laboratory have investigated the effects of TIP39 on the hypothalamo-pituitary axes using both *in-vivo* and *in-vitro* approaches (98). They found that TIP39 induces the release of AVP, adrenocorticotropic hormone (ACTH)-releasing factor, luteinizing hormone (LH)–releasing hormone, and growth hormone-releasing factor (GRF). In addition, TIP39 administration effectively suppressed the elevation of plasma AVP induced by various stimuli, including 48h water deprivation (dehydration), intraperitoneal injection of hypertonic saline (hyperosmolality), and intraperitoneal injection of polyethylene glycol (hypovolemia). These inhibitory effects were independent of changes in osmotic or hypovolemic stimulation, as evidenced by unaltered plasma sodium levels and total protein concentration following TIP39 treatment (99). These findings suggest that TIP39 positively regulates the hypothalamic-pituitary axis.

The presence of CRH-containing cells in the parvicellular subdivision of PVN and GHRH-containing cells in ARC imply that TIP39-containing neurons in SPF directly mediate hypothalamo-pituitary axes by projecting to these nuclei. Indirectly, TIP39 may influence ARC, median eminence, and parvicellular subdivision of PVN through projections to the dorsomedial nucleus, which contains a relatively high density of TIP39 (114). A possible mechanism is that TIP39 may affect glutamatergic interneurons within the PVN itself to modulate HPA axis activation, ultimately influencing neurotransmitter release from their terminals (115).

Moreover, TIP39 may promote the release of prolactin. It is well established that suckling elevates serum prolactin levels during lactation, although the underlying mechanism remains unclear. The abundance of PTH2R-expressing neurons and PTH2R-containing terminals in the arcuate nucleus suggests their role in this regulatory process, especially given the confirmed projection of TIP39 from the arcuate nucleus identified using retrograde tracers. Researchers have further explored the physiological roles of TIP39 in prolactin control circuits by antagonizing its action locally. Their findings on rats showed that a virus encoding a PTH2R antagonist significantly reduced basal prolactin levels in serum following injections into regions such as PIL and ARC. Additionally, suckling was found to increase Fos expression in PIL TIP39 neurons. This suggests that suckling may regulate prolactin release by influencing TIP39 neurons in PIL, which in turn project to ARC (116).

Current experimental studies and findings regarding the endocrine functions of TIP39 in the CNS are summarized in **Table 3**.

**Table 3:** Experimental findings for the endocrine roles of TIP39.

Hormone	*In-vivo*/*in-vitro*	Experimental approach	Key result	Proposed mechanism	References
AVP	Rats	ICV injection of TIP39 under basal/dehydration/hyperosmolality/hypovolemia	Inhibits AVP release	Central PTH2R activation in opioid-dependent pathways; osmotic/hemodynamic independent	(48, 99)
	*In-vitro* hypothalamic cell culture	Bath application of TIP39	Moderately stimulates AVP release	Mechanism remains unclear	(98)
GRF	*In-vitro* hypothalamic cell culture	Bath application of TIP39	Moderately stimulates GRF release	Mechanism remains unclear	(98)
CRH	*In-vitro* hypothalamic cell culture	Bath application of TIP39	Stimulates CRH release (300% increase)	Mechanism remains unclear	(98)
GH	Rats	ICV injection of a single large (10 mg) dose of TIP39	Markedly suppresses plasma GH levels for 3 hours	TIP39 stimulates SST release via periventricular PTH2-R, thereby inhibiting GH secretion.	(48, 117)
SST	*In-vitro* hypothalamic cell culture	Bath application of TIP39	No significant increase in release	Mechanism remains unclear; may be model-dependent	(98)
ACTH	Rats	ICV injection of TIP39	Transiently increases plasma ACTH (peaks at 10 min)	Likely mediated via hypothalamic CRH and/or AVP release	(98)
Corticosterone	Rats	PVN infusion of TIP39 ± glutamate receptor antagonists	TIP39-induced pCREB and corticosterone elevation blocked by glutamate antagonists	Acts via glutamatergic interneurons within PVN	(115)
LH	Rats	ICV or IP injection of TIP39	Transiently increases plasma LH (peaks at 10 min)	Mechanism remains unclear	(98)
Prolactin	Rats	ICV injection of PTH2R antagonist during pup exposure	Reduces basal and suckling-induced prolactin levels	TIP39 activates arcuate interneurons to inhibit tuberoinfundibular dopamine neurons, stimulating prolactin release.	(116, 118)

### 5.7 Auditory function

Studies have demonstrated that auditory inputs can stimulate TIP39 neurons; for instance, exposure to loud white noise increases c-fos expression in TIP39 neurons located in the paralemniscal area (82, 119). This suggests that processing auditory information may be an additional function of these neurons. Further supporting this hypothesis are findings from ISHH and immunocytochemistry, which have confirmed the presence of TIP39-containing fibers in various auditory structures, including the inferior colliculus (IC), ectorhinal and temporal cortices, medial geniculate body (MGB), and the dorsolateral funiculus of the spinal cord, as well as certain nuclei in SOC (72).

The posterior thalamic intralaminar complex (PTIC) appears to play a crucial role in processing auditory pressure signals. It receives weak topographic and modality-specific acoustic signals from multiple sources, including the lateral hypocolliculus cortex (LHC), the medial perigeniculate region (MPGR) (74), and regions in the temporal lobe and perinasal cortex (120). PTIC is also involved in auditory-induced fear-conditioned responses. One proposed mechanism is that PTIC transmits auditory pressure signals by projecting to the parvocellular division of PVN. Lesions to the posterior intralaminar area (PILA) and extensive damage to a broad part of the SPFp have been shown to impede glucocorticoid release in response to auditory stress (121).

Additionally, *in-vitro* studies indicate that the concentration of corticotropin-releasing factor from hypothalamic explants increases following TIP39 administration, meanwhile intracerebroventricular injection of TIP39 elevates plasma levels of adrenocorticotropic hormone (98). These findings suggest that TIP39-containing neurons in the posterior intralaminar complex of the thalamus modulate the physiological response to auditory stress by interacting with PVN. Moreover, PTIC has garnered considerable attention regarding its role in establishing learning associations between auditory stimuli. The complex integrates afferent projections from the spinal cord (122) and the superior colliculus (SC) (123), as well as projecting to the amygdala. Notably, experiments have shown that disruption of the thalamo-amygdaloid pathway results in the loss of acoustic phobic conditioned responses (72).

In summary, TIP39 from PTIC contributes significantly to acoustic fear-conditioned responses through its sophisticated projection system.

## 6 The potential role and mechanistic prospects of TIP39 in PPD

The structure, distribution, projection site, and function of TIP39 have been introduced above. We observed that TIP39 neurons project to several brain regions implicated in depression, such as the IL, POA, LHA, LS, and PVT. This suggests a possible association between TIP39 and depressive disorders. A review of the functional roles of TIP39 in the central nervous system indicates its involvement in pain modulation and its ability to alleviate fear- and stress-related anxiety. Moreover, its endocrine regulatory functions influence the HPA axis, thereby modulating stress responses. Additionally, as previously mentioned, TIP39 neurons are involved in the PIL-MPOA pathway, which is closely associated with maternal behavior. These observations lead us to focus on the potential connections between TIP39 and depression, maternal behavior, and PPD.

Nowadays, depression, especially postpartum depression, has become a serious health problem, which has aroused widespread concern. This mood disorder is characterized by persistent sadness, emotional lability, irritability, and, in severe cases, thoughts of self-harm or harm to the infant (124). The global prevalence of PPD is high, particularly in low- and middle-income countries (125). The etiology of PPD involves complex interactions among social, psychological, and biological factors. Notably, identifying reliable biomarkers is critical for understanding PPD pathophysiology and improving clinical assessment. These biomarkers are broadly categorized into hormonal, inflammatory, and biochemical classes (126). Dysregulation of specific hormones, such as allopregnanolone, prolactin, and CRH, as well as elevated proinflammatory cytokines and nutritional deficiencies, have been closely linked to PPD (127–130).

While endocrine disruption, particularly involving HPA axis–related signals, is central to PPD, the role of specific neuropeptide systems remains incompletely elucidated. In contrast to more established mediators like CRH, the function of TIP39 in PPD has been less studied. Given its specific distribution and potential role in maternal adaptation, TIP39 may represent a novel biomarker or mechanistic factor worthy of further investigation.

Therefore, this part focuses on the relationship between TIP39 and maternal behavior, depression, and postpartum depression. Although the specific molecular mechanism is not completely clear at present, we recognize the potential of TIP39 in research on the treatment of postpartum depression.

### 6.1 TIP39 and maternal behavior

TIP39 fibers and their terminations are densely distributed in the ARC and the POA of the brain. Some studies have shown that they are associated with maternal function.

Experimental studies in rodents have demonstrated that the proper functioning of the TIP39/PTH2R system is essential for the regulation and execution of maternal behaviors (131). Further exploration into the mechanisms underlying TIP39’s regulation of maternal behavior reveals that TIP39 may directly participate in the neural reflex circuits triggered by pup stimuli (132). Additionally, TIP39 is thought to modulate the expression of hormones and neuropeptides in the brain, such as prolactin, glutathione, and amylin. By influencing the expression of these substances, widely acknowledged as being associated with maternal behavior, TIP39 likely exerts its regulatory effects on maternal responses (133–135). The potential mechanisms underlying these processes are discussed in detail below.

#### 6.1.1 TIP39 may influence maternal behavior directly

Compared with the nulliparous females, the TIP39-PTH2R system has a particular function in mother rats. In female rats during lactation, TIP39 mRNA and TIP39 levels in the PIL significantly increased. Whereas this kind of change was not observed in the PVG (131). In rats, suckling can stimulate c-fos expression in TIP39 neurons within the PIL, which subsequently send projections to the MPOA. Among these, MPOA is recognized as the core regulatory region for maternal behavior. The mother rats have a series of substantial behavior changes, including lactation, licking, aggressiveness towards intruders, retrieving the pups back to the nest, and reduction in anxiety (12).

Recent studies have shown that somatosensory stimuli from pups to mothers are received and processed by PIL. Using techniques such as anterograde tract tracing, retrograde tract tracing, and immunoelectron microscopy on rat and mouse brain regions, it has been demonstrated that TIP39-positive neurons originating from the PIL project to both LSV and MPOA simultaneously, activating the inhibitory neurons in LSV and the excitatory neurons in MPOA. Among these, the neurons in LSV activated by TIP39 are GABAergic neurons. These inhibitory neurons project to the MPOA and other brain regions involved in the maternal circuitry and regulate the functions of downstream brain regions. TIP39 neurons achieve precise coordination of maternal behaviors by synchronously regulating these two circuits (136).

#### 6.1.2 TIP39 may influence maternal behavior by hormones

##### 6.1.2.1 Prolactin

TIP39 neurons are involved in the ascending sensory pathways that transmit the sucking effect to the hypothalamic center (133). TIP39 might activate interneurons in the ARC, leading to the suppression of dopamine neurons in the tuberoinfundibular pathway, ultimately promoting prolactin release from the pituitary. In PTH2-R-KO maternal mice, lactation-induced c-fos expression within the PVN undergoes a significant decrease, resulting in lower prolactin concentrations and decreased milk production (137). Prolactin has been shown not only to facilitate milk production but also to enhance maternal motivation and exert antianxiety effects during the lactation period (57, 118).

##### 6.1.2.2 Galanin

TIP39 mediates galanin expression in the POA.

Presumably, galanin neurons in the POA are involved in the regulation of maternal behavior (138). Experiments conducted on mice revealed that the targeted removal of galanin neurons caused the cessation of maternal behavior and attacks on their pups. Conversely, stimulating galanin neurons in the POA has been observed to rapidly trigger maternal care behaviors (139). Galanin neurons are possibly activated by direct neuronal inputs and prolactin. The following describes the ways in which galanin neurons are activated.

Neuronal inputs may be mediated by TIP39. Studies on the brains of mother rats demonstrated that fibers expressing TIP39, originating from the PIL, form excitatory synapses with galanin neurons in the POA. When anterograde tracer was administered into the PIL containing TIP39-expressing neurons, it confirmed that TIP39 fibers in the POA trace back to the PIL. Using immunogold electron microscopy, the glutamatergic nature of TIP39 terminals was verified by detecting glutamate at the ends of TIP39 neurons, suggesting an excitatory interaction with galanin neurons. There are two different groups of galanin neurons located respectively in the anterior commissural nucleus (ACN) and the MPOA. Suckling activates TIP39 neurons in the PIL, and these neurons project excitability separately to galanin neurons in the ACN and the MPOA, which follow-up cause the occurrence of maternal behavior (133).

Prolactin can also activate galanin neurons. Suckling activates brain regions responsible for maternal responses, and with the involvement of TIP39 neurons, this triggers the secretion of prolactin (137). Signal transducer and activator of transcription 5 (STAT5) mediate the signal transduction of prolactin. After the binding of prolactin to its receptor, STAT5 is phosphorylated and becomes pSTAT5 (140). Some studies conducted on rats have shown that galanin neurons in the ACN become pSTAT5-positive following prolactin injection, while almost no pSTAT5-positive results are detected in the MPOA galanin neurons. That means prolactin activates the ACN galanin neurons but is almost inactive to the MPOA galanin neurons (133, 134).

In addition, oxytocin and galanin are co-expressed in oxytocin neurons within the ACN of the rat brain, which may be related to maternal behavior. Oxytocin neurons in the POA seem to have a unique function in regulating maternal behavior with oxytocin administration. Oxytocin can be expressed in the ACN, PVN, and supraoptic nucleus. And only in oxytocin neurons of the ACN, oxytocin is co-expressed with galanin. However, the subcellular localization of them is different; the distribution of oxytocin is more uniform, and galanin is mostly in the soma. It is worth noting that ACN oxytocin neurons are closely related to the control of maternal behavior, which is the characteristic that differs from other oxytocin neurons (133).

##### 6.1.2.3 Amylin neurons

TIP39 fibers and fiber ends interact with amylin neurons. In the brains of mice and rats, the distribution of amylin neurons and TIP39 fibers is very similar in POA. TIP39-knockout mice exhibited a marked decrease in amylin induction. Preoptic amylin can play a role in controlling maternal behavior. Therefore, we speculate that TIP39 fiber affects maternal behavior by innervating the expression of amylin (135).

TIP39 neurons may also interact with other neurons that are involved in maternal adaptation.

For instance, TIP39 changes the levels of estradiol and progesterone during lactation by affecting CRH neurons and kisspeptin neurons, thus affecting maternal behavior. TIP39 acts on glutamatergic intermediate neurons in the PVN region. This endogenous glutamate in the PVN acts on the end of CRH neurons and activates the HPA axis (115). Administration of CRH was found to reduce the expression of Kisspeptin (Kiss1) mRNA in the ventromedial hypothalamus (VMH) and ARC, along with a decrease in Kiss1r mRNA levels in these regions (141). In rodents, Kiss1 neurons in the hypothalamus are classified into two groups: those in the ARC are referred to as Kiss1ARH, while those in the anteroventral periventricular (AVPV) and the Pe are known as Kiss1AVPV/PeN. Both can release Kisspeptin, the most effective neurotransmitter or neuropeptide to stimulate GnRH neurons (132). A decline in GnRH secretion results in reduced levels of follicle-stimulating hormone (FSH) and LH, which further reduces the levels of estradiol and progesterone during lactation. We can confirm that the levels of these two substances increase during pregnancy and decrease during lactation, while this change in the perinatal period is helpful to induce maternal behavior. On the other hand, since TIP39 fibers are abundant in the nucleus where the kiss1 neurons are located, it is speculated that TIP39 can project to kiss1 neurons. However, it should be noted that there is lacking substantive functional evidence to back up this hypothesis (131).

#### 6.1.3 Other mechanisms related to maternal behavior

In addition to the neural circuits mediated by TIP39 previously discussed, several mechanisms are related to maternal behavior, including dopamine-mediated reinforcement learning and pregnancy hormones. Dopamine plays a vital role in the emergence of maternal behavior. Dopamine neurons in the VTA emit reward-related signals, guiding the development of maternal behavior in mice through reinforcement learning (142).

Furthermore, reproductive hormones fluctuate significantly during the perinatal period, facilitating the establishment and maintenance of maternal behavior. In rats, during the perinatal period, these reproductive hormones act on specific brain regions to initiate and establish maternal behavior. These hormones also facilitate neurogenesis in the DG and the subventricular zone of the lateral ventricle, induce morphological changes in neurons within cerebral circuits, and affect glial cell plasticity. Sensory inputs from pups, including olfactory, tactile, breastfeeding, and visual stimuli, are processed by the primary sensory cortex and integrated into key nodes of the maternal behavior circuit. This integration is vital for maintaining and adjusting maternal behavior post-delivery and adapting to the needs of the offspring (143).

Post-delivery, the emergence of maternal behavior relies heavily on various sensory cues from the offspring, with oxytocin playing an essential role. For instance, when a baby’s cry stimulates auditory-related areas, the PIL connects to the PVN oxytocin neurons through TIP39 neuron projections or other mechanisms. This circuit projects downstream to the VTA, mediating maternal behavior (13).

**Figure 4:**
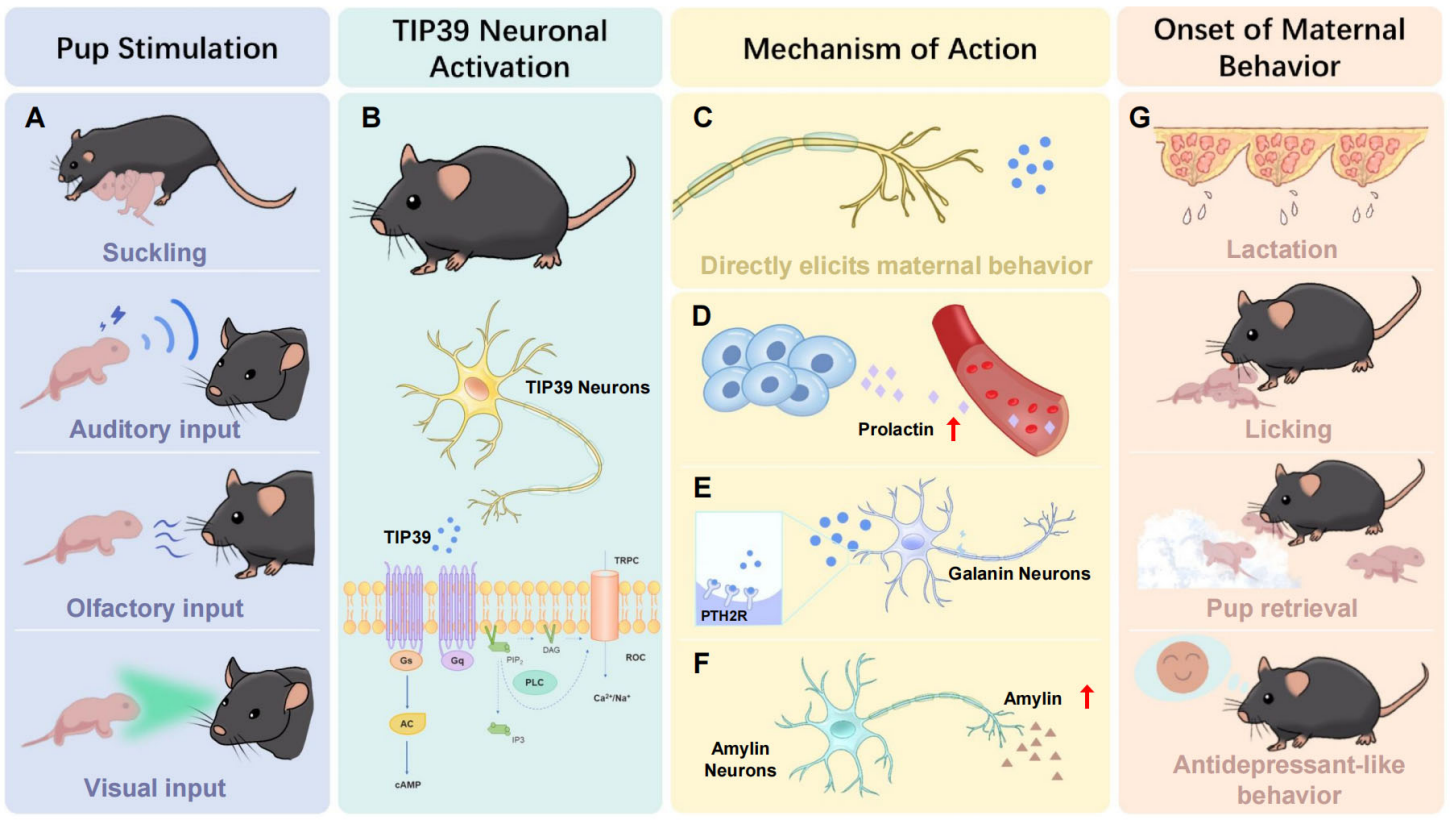
Mechanism underlying TIP39-mediated maternal behavior. **(A)** Maternal rats receive stimuli from their pups, including suckling, vocalizations, olfactory cues, and visual inputs, which are processed in the PIL. **(B)** Upon receiving these stimuli, TIP39 neurons in the PIL become activated and regulate neuronal activity in related brain regions through the release of TIP39 at their terminals. Possible signal transduction pathways for TIP39: Gs/cAMP, Gq/PLC/Ca^2+^, GPCR interaction with TRPC. **(C)** TIP39 neurons directly project to the MPOA, activating it to mediate maternal behaviors. **(D)** TIP39 transmits sensory signals to the hypothalamus, promoting prolactin release from the pituitary via regulation of the tuberoinfundibular pathway. **(E)** TIP39 released from neuronal terminals acts on PTH2R-expressing galanin neurons in the POA, forming excitatory synapses. **(F)** TIP39 released from its fibers activates amylin neurons, leading to the upregulation of amylin expression. **(G)** The MPOA, prolactin, galanin neurons, and amylin are believed to contribute to the activation and regulation of maternal behaviors. Once these circuits are activated, maternal rodents exhibit a range of maternal behaviors, including lactation, licking, retrieving pups back to the nest, and antidepressant-like behaviors.

Current research underscores the significant roles of dopamine, reproductive hormones, and neuroplasticity in establishing and maintaining maternal behavior, as well as in responding to sensory stimuli from pups. Across these mechanisms, oxytocin serves a pivotal role. TIP39 primarily acts as a bridge between receiving sensory stimulation from offspring and facilitating maternal behavior (13, 143) (see **Figure 4**).

### 6.2 The relationship between TIP39 and depression

The physiological function of TIP39 related to negative emotions has been introduced above, and here we focus on the relationship between TIP39 and depression. At present, some experiments have proved that TIP39 has antidepressant function.

The early related experiment was to administer TIP39 to male Sprague–Dawley rats by intracerebroventricular injection (ICV). Compared with the control group, TIP39 induced c-fos in five brain regions related to depression and anxiety, namely the IL, the ventrolateral region of the fornix in the lateral hypothalamus (LH, the POA, the LS, and the PV. In addition, the behavioral test results show an antidepressant effect after TIP39 administration (9, 52).

TIP39 signal transduction also plays an antidepressant role in mice after fear incubation. WT, TIP39-KO, and PTH2-R-KO male mice were given foot electric shocks as traumatic events and were evaluated by behavioral tests, such as the elevated-zero maze test, the open-field test, and the forced-swim test. It was observed that there was an augmentation in anxiety- and depression-related behaviors among mice exhibiting a deficiency in TIP39 signal transduction (95).

Besides, some experiments treated chronic unpredictable mild stress (CUMS)–induced depression rats with TIP39. In ethology, the depression-like behavior of rats was reversed after administration. Molecularly, CUMS changes the sympathetic nervous system and HPA axis, causing the disorder of the oxidant/antioxidant system and the increase of inflammatory mediators, thus causing depression. However, the administration of TIP39 reversed this change and took effect in regulating the HPA axis, oxidation process, and inflammation process, so we can infer the anti-stress activity of TIP39 (144). Apart from this, TIP39 significantly decreased the levels of glutamate and acetylcholinesterase in the brain of CUMS rats and increased the level of GABA. According to the existing animal experiments, the pathogenesis of depression caused by stress is related to the imbalance of GABA and glutamic acid levels in the brain, which is manifested by the influence of GABA receptor function, the decrease of GABA content, and the increase of extracellular glutamic acid concentration. For humans, the report shows that the concentration of GABA in plasma, cerebrospinal fluid, and brain in patients with depression also decreases. Then, it can be speculated that TIP39 may be an antidepressant by regulating the levels of GABA and glutamic acid (11, 145).

While there is currently no definitive research elucidating the role and specific mechanism of TIP39 in antidepressant effects in humans, given the promising results from animal experiments, the PTH2R receptor, in conjunction with TIP39, offers novel insights and holds broad prospects for the research on therapeutic intervention in depression.

### 6.3 TIP39 neurons project to depression-related encephalic regions

In rats, TIP39 neurons project to the IL, the POA, the LHA, the LS, and the PVT and induce the activation of c-fos in these regions, which are considered crucial factors in the manifestation of depression and anxiety (9). PTH2R is distributed in all the above encephalic regions, which is consistent with the distribution of c-fos activated cells after TIP39 administration. It illustrates that TIP39 directly acts on cells through PTH2R and activates c-fos in these regions through a similar pattern (9).

The following introduces the function of encephalic regions that receive the projection of TIP39 neurons in mediating depression.

#### 6.3.1 Infralimbic cortex

IL has been proved to be responsible for emotional response, especially negative emotions such as depression (146). For instance, after knocking down the mammalian target of rapamycin (mTOR) in IL, mice showed depressive behavior (147). In addition, in mice with depression caused by cerebral ischemia, the excitability of pyramidal neurons in layers 2, 3, and 5 of IL decreased, accompanied by the increase of rheological base current and the decrease of input resistance (148).

#### 6.3.2 Preoptic area

According to proteomic analysis of the maternal POA in rats, 12 kinds of protein increased and 6 kinds of protein decreased significantly in the mother, among which the increase of alpha-crystallin B chain (Cryab) level may help to maintain the increase of the activity of preoptic neurons. The altered proteins may be relevant to the maintenance of maternal behaviors and postpartum depression (149). In mice, GABAergic neurons in the POA can also mediate parenting behaviors, which lead to depression when they are overactivated (150). In addition, GABAergic neurons in the MPOA also mediate depression-like behaviors caused by changes in reproductive hormone levels, such as postpartum depression and perimenopausal depression (151).

#### 6.3.3 Lateral hypothalamus

In the mouse brain, there is a synaptic connection between LH neurons and the LHb, which plays an essential role in depression, and LH neurons act on LHb to produce excitatory postsynaptic potential (EPSP) in a unique discharge mode. LH-LHb synaptic enhancement appears in stress-induced depression, which may be the key to depression-like behavior (152).

#### 6.3.4 Lateral septum

A subset of GABAergic adenosine A2A receptors (A2AR)-positive neurons in LS directly project to the LHb and the dorsomedial hypothalamus (DMH) in mice, which mediates depressive symptoms. Under the pressure induction, A2AR signal transduction in neurons of the LS increased abnormally, which controlled the activity of the LS and directly projected to the LHb and the DMH through A2AR-positive neurons, causing depression-like changes. It can be seen that abnormally increased A2AR signal transduction in the LS serves as a key upstream modulator in the onset of stress-triggered depression. Therefore, A2AR antagonists may provide a scheme for antidepressants (153).

#### 6.3.5 Paraventricular thalamic nucleus

PVT projects to the NAc, amygdala, and mPFC, which are related to emotions. Among them, PVT neurons regulate the balance between neuron excitation and inhibition in the mPFC. If presynaptic disorder in PVT may lead to depression-like behavior (154). In addition, lipocalin 2 (Lcn2) was upregulated in PVT of mice with depressive behavior induced by dextran sulfate sodium (DSS), and the depressive behavior was reduced when Lcn2 was silenced, which indicated that the increase of Lcn2 in PVT was an important pathway of depression induced by DSS (155).

Although it has been confirmed that TIP39 projects into these encephalic regions related to depression, there is no clear evidence to explain what the downstream pathway of TIP39 after acting on these encephalic regions is, thus causing depression. However, the aforementioned known connections can still provide novel ideas and directions for research and applications related to TIP39.

### 6.4 Relations between TIP39 and PPD, which are known

Previous studies have shown that stress causes depression by changing the levels of neurotransmitters and glucocorticoids (156, 157). In patients with PPD, the imbalance between GABA and glutamic acid, the decrease of reproductive hormone level, and the abnormality of the HPA axis were also observed (158, 159). TIP39 fiber projects to many endocrine, marginal, and auditory areas in the brain. Based on this distribution pattern, it potentially has a function in modulating neuroendocrine activity, emotional responses, auditory processes, and pain perception (9).

Maternal-induced TIP39 may contribute to the antidepressant-like behaviors that mice exhibit when they become mothers. And PTH2R-KO mothers exhibit more depressive-like behaviors. In the case of administering exogenous TIP39, administration of TIP39 to normal mice causes antidepressant-like effects, while this change was not observed in PTH2R-KO mice. PTH2R-KO mice might be a valuable model for studying PPD, enabling the evaluation of drugs specifically designed to treat this condition (12). The following will describe the possible mechanism of postpartum anxiety and depression-like behavior caused by the abnormal TIP39/PTH2R system.

#### 6.4.1 Prolactin

As mentioned above, TIP39 mediates the production of prolactin. In PTH2R-KO female mice, the expression of c-fos induced by lactation decreased significantly in PVN, and the level of prolactin was also low. Prolactin in the first trimester is very important for the mother’s normal postpartum behavioral response. When its level is low, the possibility of postpartum anxiety increases (137, 160).

#### 6.4.2 Amylin

TIP39 neurons have been proved to activate amylin neurons, increasing the expression of amylin, which has an antidepressant effect.

TIP39 neurons within the PIL innervate the POA, stimulating amylin-producing neurons. Consequently, this leads to an upregulation of amylin expression within the POA of postnatal rats and mice. In female mice with an abnormal TIP39/PTH2R system, there was a notable reduction in the level of amylin mRNA located within the POA.

Amylin is induced following the postpartum period. It has the potential to modulate emotional and endocrine alterations in female postpartum individuals. Postpartum mother mice exhibited antidepressant-like behaviors, while mice lacking amylin and control virgin females have no this behavioral characteristic (135).

In a word, amylin has an antidepressant effect on postpartum female rats, and TIP39 is an important pathway to mediate amylin neuron activation. As a result, TIP39 and amylin emerge as promising candidates for therapeutic interventions aimed at addressing disorders associated with motherhood, such as postpartum depression.

#### 6.4.3 GABA

At present, it has been proved that women at risk of postpartum depression have low GABA levels or abnormal GABA signal transduction pathways in the perinatal period (158). In CUMS rats, the level of GABA increased after TIP39 administration (11), suggesting that TIP39 may inhibit the occurrence of postpartum depression by affecting the level of GABA in related encephalic regions.

**Figure 5:**
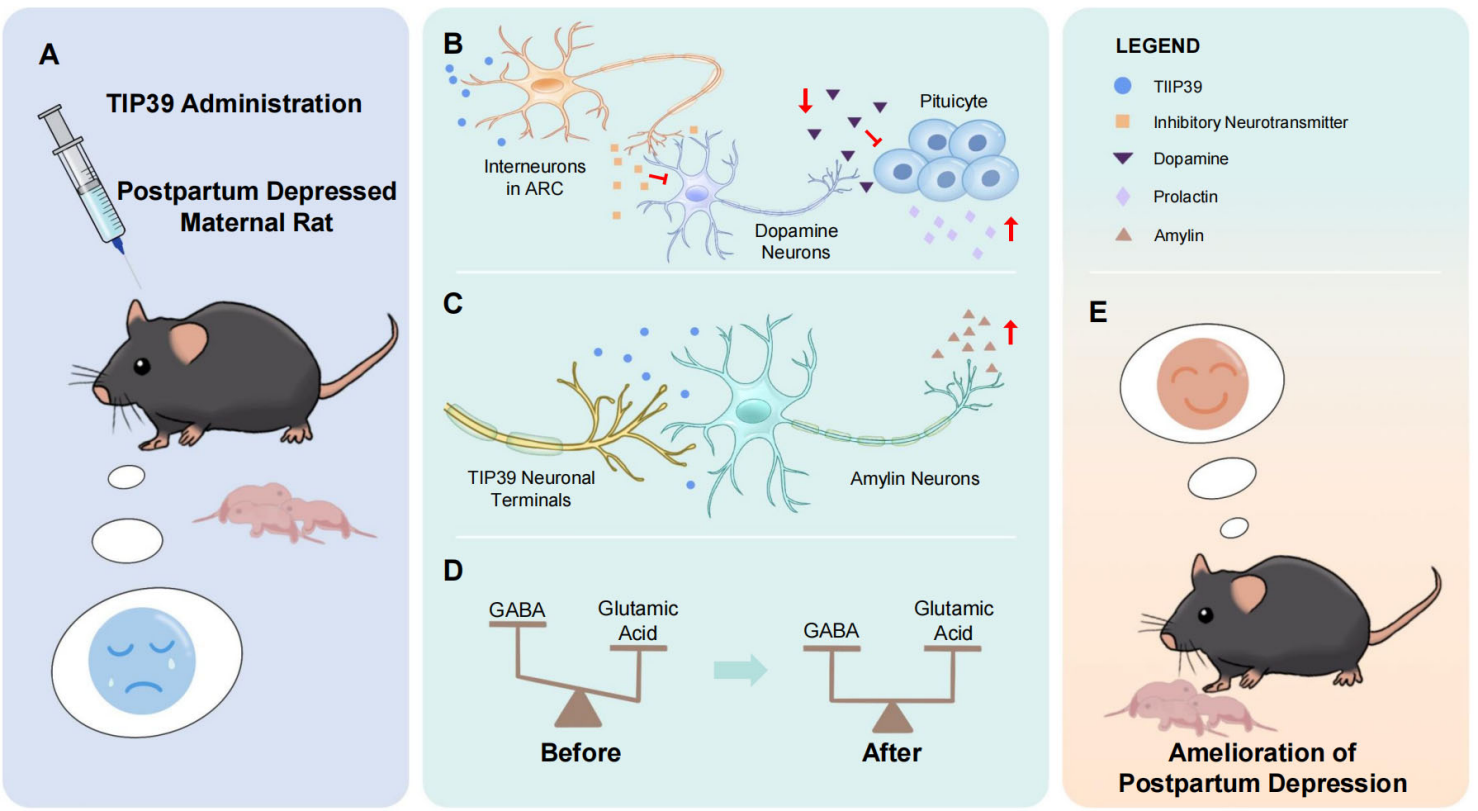
Potential mechanisms underlying TIP39 amelioration of postpartum depression. **(A)** Intrathecal injection of TIP39 in PPD-model maternal rats. **(B)** TIP39 activates inhibitory interneurons in the ARC, leading to the suppression of dopamine neurons in the tuberoinfundibular pathway. This reduces dopamine levels, thereby diminishing the inhibition of prolactin-releasing cells in the pituitary and ultimately increasing prolactin secretion. **(C)** TIP39 released from neuronal terminals activates amylin neurons, resulting in the upregulation of amylin expression. **(D)** PPD is characterized by an imbalance of neurotransmitters, particularly reduced GABA levels or disrupted GABAergic signaling, along with altered glutamatergic activity. TIP39 administration restores GABA levels, reestablishing neurotransmitter balance in the brain. **(E)** TIP39 may exert antidepressant-like effects through three mechanisms: increased prolactin secretion, upregulation of amylin expression, and neurotransmitter rebalancing, thereby alleviating symptoms of PPD.

The preceding discussion elucidates the aberrant mediation of postpartum depression pathogenesis by TIP39 dysregulation and delineates potential mechanisms through which TIP39 may ameliorate depressive phenotypes in the postpartum period (see **Figure 5**).

In addition to TIP39, neuroendocrine disorders are acknowledged as contributors to the pathophysiological mechanisms of postpartum depression, presenting numerous potential therapeutic targets. Below is a brief overview of some highly recognized mechanisms.

(1) Reproductive hormones

The dramatic fluctuations of reproductive hormones post-delivery are thought to have a function in the neurobiology underlying emotional disorders, giving rise to the “ovary-steroid-withdrawal hypothesis.” Research indicates that estrogen levels peak sharply before delivery and drop significantly below normal levels afterward, heightening the risk of PPD. In animal models, estradiol withdrawal can induce depression-like behaviors, while estrogen therapy has been shown to reduce the risk and alleviate symptoms of PPD. Hormonal changes may therefore represent a therapeutic target for addressing PPD (161, 162).

(2) Neurosteroids

Allopregnanolone, a neuroactive metabolite of progesterone, is a neurosteroid with known antidepressant and anxiolytic properties. Elevated concentrations of allopregnanolone have been linked to a decreased likelihood of developing PPD. Conversely, after antidepressant treatment, allopregnanolone levels also rise. In animal studies, allopregnanolone levels inversely correlate with depression-like behaviors. The proposed mechanism involves the modulation of GABAergic signaling, where allopregnanolone enhances the δ subunit-containing GABAARs, maintaining normal GABAergic pathways (162, 163). Brexanolone, a proprietary preparation of allopregnanolone, has been approved for treating PPD in patients aged 15 and over, marking a significant milestone in targeted PPD therapy.

(3) The hypothalamic-pituitary-adrenal (HPA) axis

Key HPA axis hormones include CRH, ACTH, and cortisol. Anomalies in the HPA axis are identified as significant contributors to PPD. From the 7th week of pregnancy, placental CRH (pCRH) secretion increases ACTH and cortisol levels, with cortisol exerting negative feedback on hypothalamic CRH but positive feedback on placental pCRH. Insufficient cortisol binding protein (CBG) levels prevent normalization of free cortisol, leading to elevated CRH, ACTH, and cortisol (86, 164). Overactivation of the HPA axis results in reduced stress responsiveness and chronic cortisol elevation due to impaired feedback mechanisms. PPD patients often exhibit high cortisol levels, weakened cortisol arousal response (CAR), and a flattened diurnal cortisol slope, indicating HPA axis imbalance. Hyper glucocorticoids exposure impacts the mPFC, amygdala, and hippocampus, impairing emotional processing and environmental adaptation (156, 164).

(4) Arginine vasopressin

AVP functions as a hormone in systemic circulation, influencing vascular smooth muscle and renal water reabsorption. In the CNS, AVP acts on V1a and V1b receptors, involved in circadian rhythm, body temperature, social behavior, and emotional stress response regulation. AVP enhances acute and chronic stress responses via the HPA axis, potentially contributing to the development of stress-induced depression, making it a potential therapeutic target. Additionally, AVP influences key physiological processes, including glucose metabolism, circadian rhythm, and cardiovascular function (165).

### 6.5 Summary and future perspectives

This section highlights the critical role of the TIP39/PTH2R system in linking maternal physiology with emotional state. Anatomically, TIP39 signaling in areas like the ARC and POA is essential for organizing maternal behavior. Functionally, the system produces antidepressant-like effects, potentially by balancing neurotransmitters and influencing mood-related brain circuits. A key finding is that TIP39 promotes adaptive responses after birth, and enhancing its activity could help improve symptoms related to PPD, possibly by regulating key factors such as prolactin, amylin, and GABA.

Unlike the widely acting systems of estrogen, CRH, and AVP, the TIP39/PTH2R pathway operates with greater specificity. Its predominant CNS localization and close link to PPD offer a unique window into the disorder’s neurobiological basis.

The TIP39/PTH2R system uniquely connects the biological needs of motherhood with emotional control in animal models. A critical gap, however, is the current lack of direct evidence for TIP39’s role in human PPD. Its positive role in preclinical studies offers a promising direction, but future work must not only clarify the precise cellular mechanisms in animal models but also prioritize investigations in human populations. A deeper understanding of how to leverage this natural system, and whether these mechanisms are conserved in humans, is vital for developing new treatment approaches that target the connection between hormones and mood.

## 7 Limitations

Since the report of its initial discovery in 1997 (4), the literature surrounding TIP39 has grown after systemic delivery. This review cites documents from 1999 to 2025, acknowledging its inherent limitations and possible omissions.

This review comprises molecular characterization of TIP39 as well as its receptor PTH2R, TIP39 expression in the brain, and central regulatory mechanisms that TIP39 involves in. Existing animal experiments, data, and analysis reviewed here allow the assumption that there might be a possible tie between TIP39 and PPD. TIP39 fibers reach brain regions linked to depression, including the IL, the POA, the LH, the LS, the PVN, the PFC, and so forth. (see 4.3 and **Table 2**). TIP39 has also been proved to influence maternal behaviors directly or indirectly. Yet, to forge a definite bond between them, many unanswered questions remain.

Additionally, the pattern of TIP39 projection in the MPL and signs of catabolism of TIP39 still need to be looked for. Owing to the need for further elucidation of its mechanisms, research on TIP39 has not yet entered clinical trials, and data for this section is currently unavailable. There is still a long way to go from lab to clinic. Only after fully understanding its downward path can the most relevant loop of PPD be identified, and then more targeted and specific studies can be carried out. Finally, the scarcity of validated commercial reagents poses a methodological constraint, necessitating reliance on custom-generated antibodies. Establishing standardized tools is therefore imperative to facilitate a reproducible future.

## 8 Conclusions and perspective

**Figure 6:**
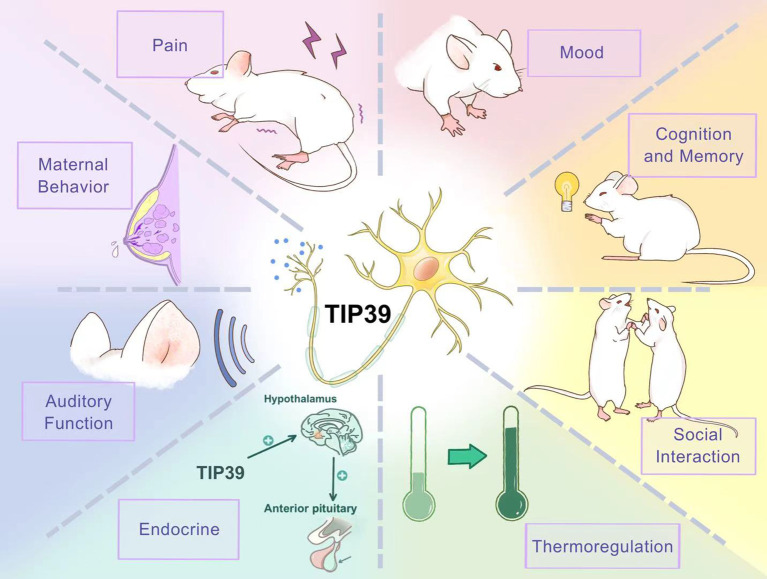
Overview of the function of TIP39 in the brain, including: 1) Pain. 2) Mood regulation. 3) Cognition and memory. 4) Social interaction. 5) Thermoregulation. 6) Endocrine. 7) Auditory Function. 8) Maternal behavior. Representative icons are shown.

The member of the parathyroid-related peptide family, TIP39, forms a unique neuropeptide-receptor system with PTH2R. The system participates broadly in central regulation, including nociceptive information processing, affective processing, cognition and memory, neuroendocrine actions, auditory system modulation, and central reproductive regulation (see graphic abstract in **Figure 6**).

Despite of its constraints, the review comes up with its view of TIP39’s potential in diagnostic and therapeutic applications in postpartum depression. TIP39’s effects in the central nervous system support the notion that its antidepressant activities and pro-maternal effects are mediated, at least to some extent, via upregulated GABAergic neuron, decreased glutamate neurotransmission and hormone regulation. Significant roles TIP39 played in endocrine regulation mainly consist of increased ACTH and oxytocin as well as decreased AVP. To affirm the possible connection between TIP39 and PPD, further proof-of-principle studies *in vitro* and *in vivo* in small animal models need to be carried out.

To make further translational claims after animal and *in-vitro* studies, preclinical and clinical trials are needed. For preclinical state, finding target validation in human cell lines and testing toxicology and pharmacokinetics of TIP39 are necessary. Assessing the efficacy of TIP39 modulation in reversing or delaying the progression of PPD symptoms in animal models is also acquired. Then, for clinical phase, initiate Phase I clinical trials to evaluate the safety and tolerability of the selected therapeutic candidate in healthy volunteers and patients with PPD should be the first stage. In Phase II Clinical Trials, larger scale studies are to be conducted to determine the efficacy of the therapeutic candidate in a broader patient population with PPD, and validated clinical outcome measures can be used to assess the therapeutic effect on PPD symptoms. Entering Phase III Clinical Trials, it is expected to monitor long-term outcomes and side effects to ensure the therapeutic is both effective and safe for long-term use.
