# Supplementary material for: Neuropeptide TIP39 as a pluripotent brain molecule and its potential functions in postpartum depression
Source: Front Endocrinol (Lausanne). 2026 Jan 8;16:1674213. doi: 10.3389/fendo.2025.1674213 (PMC12823539; doi:10.3389/fendo.2025.1674213)
Supplement: Supplementary file 1 [file DataSheet1.pdf]

Search strategy - PRISMA

PubMed		Total
#1	(((((((((“tuberoinfundibular peptide 39”[MeSH Terms]) OR (“pth2 protein, zebrafish”[MeSH Terms])) OR (“parathyroid hormone 2 (1-34), zebrafish”[MeSH Terms])) OR (“PTH2 protein, human”[MeSH Terms])) OR (“TIP39”[Title/Abstract])) OR (“PTH2”[Title/Abstract])) OR (“Tuberoinfundibular peptide of 39 residues”[Title/Abstract])) OR (“tuberoinfundibular peptide 39”[Title/Abstract])) OR (“Parathyroid hormone 2”[Title/Abstract])) OR (“Parathyroid hormone family” [Title/Abstract]))	298
#2	((((Receptor, Parathyroid Hormone, Type 2[MeSH Terms]) OR (PTH2R [Title/Abstract])) OR (Receptor, Parathyroid Hormone, Type 2[Title/Abstract])) OR (Parathyroid hormone 2 receptor*[Title/Abstract]))	117
#3	(((((((((((((((((((((Nervous System Physiological Phenomena[MeSH Terms]) OR (Stress, Physiological[MeSH Terms])) OR (Baroreflex[MeSH Terms])) OR (Sensation[MeSH Terms])) OR (Hearing[MeSH Terms])) OR (Pain[MeSH Terms])) OR (Proprioception[MeSH Terms])) OR (Smell[MeSH Terms])) OR (Thermosensing[MeSH Terms])) OR (Vision, Ocular[MeSH Terms])) OR (Social Interaction[MeSH Terms])) OR (brain function[Title/Abstract])) OR (stress[Title/Abstract])) OR (reflex[Title/Abstract])) OR (Hearing[Title/Abstract])) OR (Auditory function[Title/Abstract])) OR (Pain[Title/Abstract])) OR (Proprioception[Title/Abstract])) OR (Smell[Title/Abstract])) OR (Thermosensing[Title/Abstract])) OR (Vision, Ocular[Title/Abstract])) OR (Social Interaction[Title/Abstract]))	3,403,385
#4	(((((((((((((((((((Maternal Behavior[MeSH Terms]) OR (Lactation[MeSH Terms])) OR (Behavior, Maternal[Title/Abstract]))) OR (Behavior*, Maternal[Title/Abstract]))) OR (Maternal Behaviors[Title/Abstract]))) OR (Maternal Care Pattern*[Title/Abstract]))) OR (Care Pattern, Maternal[Title/Abstract]))) OR (Care Pattern*, Maternal[Title/Abstract]))) OR (Maternal Care Pattern*[Title/Abstract]))) OR (Pattern, Maternal Care[Title/Abstract]))) OR (Patterns, Maternal Care[Title/Abstract]))) OR (Maternal Patterns of Care[Title/Abstract]))) OR (Maternal Pattern of Care[Title/Abstract]))) OR (Lactation[Title/Abstract])) OR (Milk Secretion[Title/Abstract])) OR (Milk Secretions[Title/Abstract])) OR (Lactation, Prolonged[Title/Abstract])) OR (Lactations, Prolonged[Title/Abstract])) OR (Prolonged Lactation[Title/Abstract])) OR (Prolonged Lactations[Title/Abstract]))	127,296
#5	((((((((((((((((Depression, Postpartum[MeSH Terms]) OR (Postpartum Depression[Title/Abstract])) OR (Post-Natal	14,136

	Depression[Title/Abstract])) OR (Depression, Post-Natal[Title/Abstract])) OR (Post Natal Depression[Title/Abstract])) OR (Post-Partum Depression[Title/Abstract])) OR (Depression, Post-Partum[Title/Abstract])) OR (Post Partum Depression[Title/Abstract])) OR (Postnatal Depression[Title/Abstract])) OR (Depression, Postnatal[Title/Abstract])) OR (Postnatal Dysphoria[Title/Abstract])) OR (Dysphoria, Postnatal[Title/Abstract])) OR (Postpartum Dysphoria[Title/Abstract])) OR (Dysphoria, Postpartum[Title/Abstract])) OR (Post-Partum Dysphoria[Title/Abstract])) OR (Dysphoria, Post-Partum[Title/Abstract])) OR (Post Partum Dysphoria[Title/Abstract])) OR (Post-Natal Dysphoria[Title/Abstract])) OR (Dysphoria, Post-Natal[Title/Abstract])) OR (Post Natal Dysphoria[Title/Abstract]))	
#6	#1 AND #2 AND #3 AND #4 AND #5	290

**Embase**		**Total**
#1	'tuberoinfundibular peptide 39'/exp OR 'tip39':ab,ti OR 'pth2':ab,ti OR 'tuberoinfundibular peptide of 39 residues':ab,ti OR 'tuberoinfundibular peptide 39':ab,ti OR 'parathyroid hormone 2':ab,ti OR 'parathyroid hormone family':ab,ti	292
#2	'parathyroid hormone receptor 2'/exp OR 'pth2r':ab,ti OR 'receptor, parathyroid hormone, type 2':ab,ti OR 'parathyroid hormone 2 receptor':ab,ti OR 'parathyroid hormone receptor 2':ab,ti	173
#3	'nervous system function'/exp OR 'physiological stress'/exp OR 'pressoreceptor reflex'/exp OR 'sensation'/exp OR 'hearing'/exp OR 'pain'/exp OR 'proprioception'/exp OR 'odor'/exp OR 'temperature sense'/exp OR 'vision'/exp OR 'social interaction'/exp OR 'nervous system function':ab,ti OR 'brain function':ab,ti OR 'physiological stress':ab,ti OR 'stress':ab,ti OR 'reflex':ab,ti OR 'hearing':ab,ti OR 'auditory function':ab,ti OR 'pain':ab,ti OR 'proprioception':ab,ti OR 'smell':ab,ti OR 'thermosensing':ab,ti OR 'vision, ocular':ab,ti OR 'social interaction':ab,ti	6,370,976
#4	'maternal behavior'/exp OR 'lactation'/exp OR ‘behavior, maternal’:ab,ti OR ‘behavior*,maternal’:ab,ti OR ‘maternal behaviors’:ab,ti OR ‘maternal care pattern*’:ab,ti OR ‘care pattern, maternal’:ab,ti OR ‘care pattern*, maternal’:ab,ti OR ‘maternal care pattern*’:ab,ti OR ‘pattern, maternal care’:ab,ti OR ‘patterns, maternal care’:ab,ti OR ‘maternal patterns of care’:ab,ti OR ‘maternal pattern of care’:ab,ti OR ‘lactation’:ab,ti OR ‘milk secretion’:ab,ti OR ‘milk secretions’:ab,ti OR ‘lactation, prolonged’:ab,ti OR ‘lactations,	101,351

	prolonged':ab,ti OR 'prolonged lactation':ab,ti OR 'prolonged lactations':ab,ti	
#5	'postnatal depression'/exp OR 'Postpartum Depression':ab,ti OR 'Post-Natal Depression':ab,ti OR 'PPD':ab,ti OR 'Depression, Post-Natal':ab,ti OR 'Post Natal Depression':ab,ti OR 'Post-Partum Depression':ab,ti OR 'Depression, Post-Partum':ab,ti OR 'Post Partum Depression':ab,ti OR 'Postnatal Depression':ab,ti OR 'Depression, Postnatal':ab,ti OR 'Postnatal Dysphoria':ab,ti OR 'Dysphoria, Postnatal':ab,ti OR 'Postpartum Dysphoria':ab,ti OR 'Dysphoria, Postpartum':ab,ti OR 'Post-Partum Dysphoria':ab,ti OR 'Dysphoria, Post-Partum':ab,ti OR 'Post Partum Dysphoria':ab,ti OR 'Post-Natal Dysphoria':ab,ti OR 'Dysphoria, Post-Natal':ab,ti OR 'Post Natal Dysphoria':ab,ti	38,663
#6	#1 AND (#2 OR #3 OR #4 OR #5)	139

**Web of Science**		**Total**
#1	((((((((((((((TS=(tuberoinfundibular peptide 39)) OR TS=(pth2 protein, zebrafish)) OR TS=(parathyroid hormone 2 (1-34), zebrafish)) OR TS=(PTH2 protein, human)) OR TI=(TIP39)) OR TI=(PTH2)) OR TI=(Tuberoinfundibular peptide of 39 residues)) OR TI=(tuberoinfundibular peptide 39)) OR TI=(Parathyroid hormone 2)) OR TI=(Parathyroid hormone family)) OR AB=(TIP39)) OR AB=(PTH2)) OR AB=(Tuberoinfundibular peptide of 39 residues)) OR AB=(tuberoinfundibular peptide 39)) OR AB=(Parathyroid hormone 2)) OR AB=(Parathyroid hormone family))	10,554
#2	((((((TS=(Receptor, Parathyroid Hormone, Type 2)) OR TI=(PTH2R)) OR TI=(Receptor, Parathyroid Hormone, Type 2)) OR TI=(Parathyroid hormone 2 receptor)) OR AB=(PTH2R)) OR AB=(Receptor, Parathyroid Hormone, Type 2)) OR AB=(Parathyroid hormone 2 receptor))	2,192
#3	(((((((((((((((((((((((((((((((TS=(Nervous System Physiological Phenomena)) OR TS=(Stress, Physiological)) OR TS=(Baroreflex)) OR TS=(Sensation)) OR TS=(Hearing)) OR TS=(Pain)) OR TS=(Proprioception)) OR TS=(Smell)) OR TS=(Thermosensing)) OR TS=(Vision, Ocular)) OR TS=(Social Interaction)) OR TI=(brain function)) OR TI=(stress)) OR TI=(reflex)) OR TI=(Hearing)) OR TI=(Auditory function)) OR TI=(Pain)) OR TI=(Proprioception)) OR TI=(Smell)) OR TI=(Thermosensing)) OR TI=(Vision, Ocular)) OR TI=(Social Interaction)) OR AB=(brain function)) OR AB=(stress)) OR AB=(reflex)) OR AB=(Hearing)) OR AB=(Auditory function)) OR AB=(Pain)) OR AB=(Proprioception)) OR AB=(Smell)) OR AB=(Thermosensing)) OR AB=(Vision, Ocular)) OR AB=(Social Interaction))	3,585,040
#4	(((((((((((((((((((TS=(Maternal Behavior)) OR TS=(Lactation)) OR TI=(Maternal Behavior*)) OR TI=(Maternal Care Pattern*)) OR TI=(Care	105,144

	Pattern*, Maternal)) OR TI=(Pattern*, Maternal Care)) OR TI=(Maternal Pattern* of Care)) OR TI=(Lactation)) OR TI=(Milk Secretion*)) OR TI=(Lactation*, Prolonged)) OR TI=(Prolonged Lactation*)) OR AB=(Maternal Behavior*)) OR AB=(Maternal Care Pattern*)) OR AB=(Care Pattern*, Maternal)) OR AB=(Pattern*, Maternal Care)) OR AB=(Maternal Pattern* of Care)) OR AB=(Lactation)) OR AB=(Milk Secretion*)) OR AB=(Lactation*, Prolonged)) OR AB=(Prolonged Lactation*))	
#5	((((((((((((((((((((((((((((((((((((((TS=(Depression, Postpartum)) OR TI=(Postpartum Depression)) OR TI=(Post-Natal Depression)) OR TI=(Depression, Post-Natal)) OR TI=(Post Natal Depression)) OR TI=(Post-Partum Depression)) OR TI=(Depression, Post-Partum)) OR TI=(Post Partum Depression)) OR TI=(Postnatal Depression)) OR TI=(Depression, Postnatal)) OR TI=(Postnatal Dysphoria)) OR TI=(Dysphoria, Postnatal)) OR TI=(Postpartum Dysphoria)) OR TI=(Dysphoria, Postpartum)) OR TI=(Post-Partum Dysphoria)) OR TI=(Dysphoria, Post-Partum)) OR TI=(Post Partum Dysphoria)) OR TI=(Post-Natal Dysphoria)) OR TI=(Dysphoria, Post-Natal)) OR TI=(Post Natal Dysphoria)) OR AB=(Postpartum Depression)) OR AB=(Post-Natal Depression)) OR AB=(Depression, Post-Natal)) OR AB=(Post Natal Depression)) OR AB=(Post-Partum Depression)) OR AB=(Depression, Post-Partum)) OR AB=(Post Partum Depression)) OR AB=(Postnatal Depression)) OR AB=(Depression, Postnatal)) OR AB=(Postnatal Dysphoria)) OR AB=(Dysphoria, Postnatal)) OR AB=(Postpartum Dysphoria)) OR AB=(Dysphoria, Postpartum)) OR AB=(Post-Partum Dysphoria)) OR AB=(Dysphoria, Post-Partum)) OR AB=(Post Partum Dysphoria)) OR AB=(Post-Natal Dysphoria)) OR AB=(Dysphoria, Post-Natal)) OR AB=(Post Natal Dysphoria))	21,911
#6	#1 AND #2 AND (#3 OR #4 OR #5)	124

**Google Scholar**		**Total**
#1	Title: “tuberoinfundibular peptide 39” OR “pth2 protein” OR “parathyroid hormone 2 (1-34)” OR “TIP39” OR “PTH2” OR “Tuberoinfundibular peptide of 39 residues” OR “tuberoinfundibular peptide 39” OR “Parathyroid hormone 2” OR “Parathyroid hormone family”	681
#2	Title: “Receptor, Parathyroid Hormone, Type 2” OR “PTH2R” OR “Receptor, Parathyroid Hormone, Type 2” OR “Parathyroid hormone 2 receptor”	217
#3	Title: “Nervous System Physiological Phenomena” OR “Stress, Physiological” OR “Baroreflex” OR “Sensation” OR “Hearing” OR “Pain” “Proprioception” OR “Smell” OR “Thermosensing” OR “Vision, Ocular” OR “Social Interaction” OR “brain function” OR “stress” OR “reflex” OR	481,000

	"Hearing" OR "Auditory function" OR "Proprioception" OR "Smell" OR "Thermosensing" OR "Vision, Ocular" OR "Social Interaction"	
#4	Title: "Maternal Behavior" OR "Lactation" OR "Behavior, Maternal" OR "Behavior, Maternal" OR "Maternal Behaviors" OR "Maternal Care Pattern" OR "Care Pattern, Maternal" OR "Care Pattern, Maternal" OR "Maternal Care Pattern" OR "Pattern, Maternal Care" OR "Patterns, Maternal Care" OR "Maternal Patterns of Care" OR "Maternal Pattern of Care" OR "Lactation" OR "Milk Secretion" OR "Milk Secretions" OR "Lactation, Prolonged" OR "Lactations, Prolonged" OR "Prolonged Lactation" OR "Prolonged Lactations"	31,700
#5	Title: "Depression, Postpartum" OR "Postpartum Depression" OR "Post-Natal Depression" OR "Depression, Post-Natal" OR "Post Natal Depression" OR "Post-Partum Depression" OR "Depression, Post-Partum" OR "Postnatal Depression" OR "Depression, Postnatal" OR "Postnatal Dysphoria" OR "Dysphoria, Postnatal" OR "Postpartum Dysphoria" OR "Dysphoria, Postpartum" OR "Post-Partum Dysphoria" OR "Dysphoria, Post-Partum" OR "Post Partum Dysphoria" OR "Post-Natal Dysphoria" OR "Dysphoria, Post-Natal" OR "Post Natal Dysphoria"	34,500
#6	("tuberoinfundibular peptide 39" OR "pth2 protein" OR "parathyroid hormone 2 (1-34)" OR "TIP39" OR "PTH2" OR "Tuberoinfundibular peptide of 39 residues" OR "tuberoinfundibular peptide 39" OR "Parathyroid hormone 2" OR "Parathyroid hormone family") AND ("Receptor, Parathyroid Hormone, Type 2" OR "PTH2R" OR "Receptor, Parathyroid Hormone, Type 2" OR "Parathyroid hormone 2 receptor") AND ("Nervous System Physiological Phenomena" OR "Stress, Physiological" OR "Baroreflex" OR "Sensation" OR "Hearing" OR "Pain" "Proprioception" OR "Smell" OR "Thermosensing" OR "Vision, Ocular" OR "Social Interaction" OR "brain function" OR "stress" OR "reflex" OR "Hearing" OR "Auditory function" OR "Proprioception" OR "Smell" OR "Thermosensing" OR "Vision, Ocular" OR "Social Interaction") AND("Maternal Behavior" OR "Lactation" OR "Behavior, Maternal" OR "Behavior, Maternal" OR "Maternal Behaviors" OR "Maternal Care Pattern" OR "Care Pattern, Maternal" OR "Care Pattern, Maternal" OR "Maternal Care Pattern" OR "Pattern, Maternal Care" OR "Patterns, Maternal Care" OR "Maternal Patterns of Care" OR "Maternal Pattern of Care" OR "Lactation" OR "Milk Secretion" OR "Milk Secretions" OR "Lactation, Prolonged" OR "Lactations, Prolonged" OR "Prolonged Lactation" OR "Prolonged Lactations") AND ("Depression, Postpartum" OR "Postpartum Depression" OR "Post-Natal Depression" OR "Depression, Post-Natal" OR "Post Natal Depression" OR "Post-Partum Depression" OR "Depression, Post-Partum" OR "Postnatal Depression" OR "Depression, Postnatal" OR "Postnatal Dysphoria" OR "Dysphoria, Postnatal" OR "Postpartum Dysphoria" OR "Dysphoria, Postpartum"	584

	OR “Post-Partum Dysphoria” OR “Dysphoria, Post-Partum” OR “Post Partum Dysphoria” OR “Post-Natal Dysphoria” OR “Dysphoria, Post-Natal” OR “Post Natal Dysphoria”)	

**ScienceDirect**		**Total**
#1	((((((“tuberoinfundibular peptide 39”) OR (“pth2 protein”)) OR (TIP39)) OR (PTH2)) OR (“Tuberoinfundibular peptide of 39 residues”)) OR (“Parathyroid hormone 2”)) OR (“Parathyroid hormone family”))	1,006
#2	(((“Receptor, Parathyroid Hormone, Type 2”) OR (PTH2R)) OR (“Parathyroid hormone 2 receptor”))	289
#3	(((((((“Stress, Physiological”) OR (Sensation)) OR (“Auditory function”)) OR (Pain)) OR (Thermosensing)) OR (“Social Interaction”)) OR (“brain function”)) OR (reflex))	3071139 （years:1995-2025）
#4	((((((“Maternal Behavior”) OR (“Behavior, Maternal”)) OR (Lactation)) OR (“Care Pattern, Maternal”)) OR (“Maternal Care Pattern”)) OR (“Milk Secretion”)) OR (“Lactation, Prolonged”))	145,123
#5	(((((((“Depression, Postpartum”) OR (“Postpartum Depression”)) OR (“Postnatal Depression”)) OR (“Postpartum Depression”)) OR (“Postnatal Depression”)) OR (“Postnatal Dysphoria”)) OR (“Postpartum Dysphoria”)) OR (“Postnatal Dysphoria”))	16,138
#6	(("Tuberoinfundibular peptide of 39 residues") OR ("parathyroid hormone 2") OR ("Parathyroid hormone 2 receptor") OR (TIP39)) AND ((brain) OR ("Central Nervous System") OR ("Maternal Behaviour") OR ("Postpartum Depression") OR ("Postpartum Dysphoria"))	238
